# Synthesis, liquid crystalline behaviour and structure–property relationships of 1,3-bis(5-substituted-1,3,4-oxadiazol-2-yl)benzenes

**DOI:** 10.3762/bjoc.16.17

**Published:** 2020-01-31

**Authors:** Afef Mabrouki, Malek Fouzai, Armand Soldera, Abdelkader Kriaa, Ahmed Hedhli

**Affiliations:** 1 Laboratory of Molecular Organic Chemistry, National Higher Engineering School of Tunis, 5 avenue Taha Hussein, Montfleury, 1089, Tunis, Tunisia; 2 LR99ES16 Physics Laboratory of Soft Matter and Electromagnetic Modelling, University of Tunis El Manar, 2092, Tunis, Tunisia; 3 Department of Chemistry, Quebec Center for Functional Materials, University of Sherbrooke, Sherbrooke, Québec, J1K 2R1, Canada

**Keywords:** dipole moment, fluorine, liquid crystal, oxadiazole

## Abstract

Two series containing 1,3-bis(1,3,4-oxadiazol-2-yl)benzene as a rigid core (RC) and alkyl or perfluoroalkyl as terminal chains were synthesized and characterized. Liquid crystal properties of the synthesized compounds have been investigated by polarizing optical microscopy, differential scanning calorimetry and X-ray diffraction techniques. Conformation effects of the synthesized products on the dipole moments were also investigated.

## Introduction

Liquid-crystalline (LC) materials have been known for over a century [1]. It is clear that architecture and functionalization are essential aspects in molecular engineering of liquid crystals [2]. The introduction of fluorine atoms in the molecular structure presents a successful strategy to control the liquid crystal proprieties. The element Fluorine presents the highest electronegativity, the lowest polarizability and a small radius. When bonded to carbon, it forms the strongest single bond in organic chemistry [3]. The C–F bond is highly polarized and this polarity inhibits the lone pair donation from fluorine, making this element a weak coordinator. These properties are the basis for the unique properties of perfluoroalkylated compounds such as high viscosity, high density, high chemical stability, low surface tension, low dielectric constants and low refractive index [4]. The usefulness of these properties makes fluorine an element of choice for the enhancement of promising properties, remained inaccessible otherwise. However, the combination of small size and high polarity of the fluorine atom leads to a subtle modification of properties such as melting point, mesophase morphology, transition temperatures, optical anisotropy, dielectric anisotropy, and visco-elasticity [5–10]. Therefore, many fluorinated liquid crystals have been prepared, and the fluoro-substitution effect has been well studied, especially in the fluoroaromatic derivatives [11–13].

Additionally, the mesomorphic properties of liquid crystals depend strongly on the nature of the terminal chains that are present. A terminal perfluorocarbon chain present in a LC molecule causes stiffening, which generates a lamellar packing and thus contributes to smectic phase stability [14]. It was reported that even simple *n*-alkanes containing a fluorocarbon block produces smectic phases [15–19]. Some molecules having only a single aromatic ring and fluorinated tail show smectic phases, while their hydrocarbon counterparts are non-mesomorphic [20].

On the other hand, many heterocyclic-based liquid crystals have been designed and then synthesised due to their large scope of applications [21]. In this context, the 1,3,4-oxadiazole group was of a particular interest from a synthetic viewpoint, considering the numerous ways to introduce this group into a molecule [22–23]. So, it was established that the introduction of a 1,3,4-oxadiazole ring as terminal group on a biphenyl provides liquid crystalline materials [24–25]. However, when the oxadiazole unit is incorporated in the aromatic core, the obtained compounds do not exhibit mesomorphism, only crystal–isotropic transitions were observed [26]. The absence of mesophases is mainly due to the strong bend (134°) between the aromatic rings, which disturbs the linear shape of the whole molecule [27].

Herein we describe the synthesis and characterization of two series of hydro- and fluorocarbonated 1,3-bis(1,3,4-oxadiazol-2-yl)benzenes. Structure–property relationships of the obtained compounds were investigated.

## Results and Discussion

### Synthesis

**Scheme 1:**
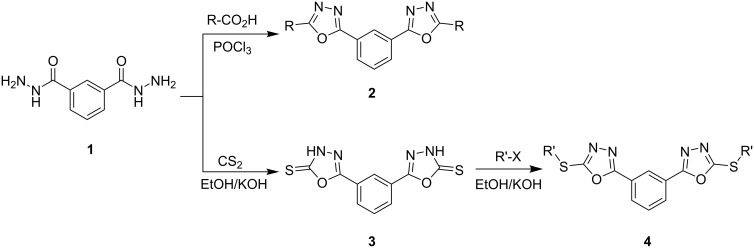
Synthesis of oxadiazole derivatives **2** and **4**.

When benzene-1,3-dicarbohydrazide [28] (**1**) was allowed to react with hydro- or perfluorocarboxylic acids in the presence of phosphorus oxychloride according to standard methods [29], oxadiazole derivatives **2** were obtained (Scheme 1). On the other hand, we converted compound **1** into sulfanyloxadiazole derivatives **4** by treatment with carbon disulfide and subsequent alkylation of the obtained intermediate **3** (Scheme 1).

Table 1 shows the yields and melting points of the synthesized compounds **2** and **4**.

**Table 1:** Obtained oxadiazole derivatives **2** and **4**.

Compound		R / R’	X	Yield (%)	Mp (°C)

**2**	**a**	C_6_F_13_	–	65	106
	**b**	C_7_F_15_	–	61	120
	**c**	*n*-C_9_H_19_	–	75	83
	**d**	C_6_H_5_	–	72	74
**4**	**a**	C_6_F_13_C_2_H_4_	I	74	128
	**b**	C_8_F_17_C_2_H_4_	I	66	138
	**c**	*n*-C_4_H_9_	Br	70	69
	**d**	*n*-C_12_H_25_	Br	72	88

**Scheme 2:**
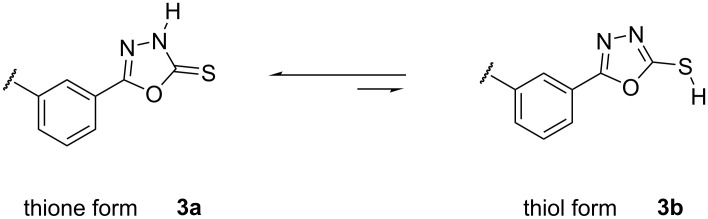
Tautomeric equilibrium of compound **3**.

The tautomeric equilibrium of compound **3** is illustrated in Scheme 2. On the basis of FTIR data, it has been concluded that in solution, the equilibrium is shifted to the thione form **3a** rather than the thiol one **3b**. The observed IR absorptions at 3387 cm^−1^ (ν_N-H_) and 1263 cm^−1^ (ν_C=S_) and the absence of absorptions in the 2600–2550 cm^−1^ region (ν_S-H_) support the preference for the thione form in solution. This latter is obviously more solvated than thiol.

### Liquid crystal properties

The structure of compounds **2** and **4** is constituted by a rigid core (three aromatic rings) to which are attached the terminal chains. Based on this structure, some liquid crystalline mesophases were expected. Differential scanning calorimetry (DSC), polarized optical microscopy (POM) and X-ray diffraction pattern analysis were used to investigate this behaviour.

#### DSC measurements

Phase transition temperature (*T*_t_), melting temperatures (*T*_i_) and enthalpy changes (∆*H*_t_) of compounds **2** and **4** are summarized in Table 2.

**Table 2:** DSC thermograms data of compounds **2** and **4**.

Compound		Heating				Cooling				*T*_i_ (°C)
				
		*T*_t_ (°C)		*∆H*_t_ (J/g)		*T*_t_ (°C)		*∆H*_t_ (J/g)		

**2a**		115.50		−48.49		85.42		41.61		116.91
**2b**		115.80		−2.15		103.85		48.13		126.13
		125.11		−50.55						
**4a**		129.85		−63.76		91.63		1.89		130.81
						98.92		48.33		
**4b**		118.00		−8.39		96.51		7.21		139.92
		138.41		−41.66		121.13		36.28		
**4d**		77.10		−133.43		49.24		95.09		78.31

Based on the data given in Table 2, we found that fluorinated compounds present supplementary transition temperatures compared with the hydrocarbonated analogues.

**Figure 1:**
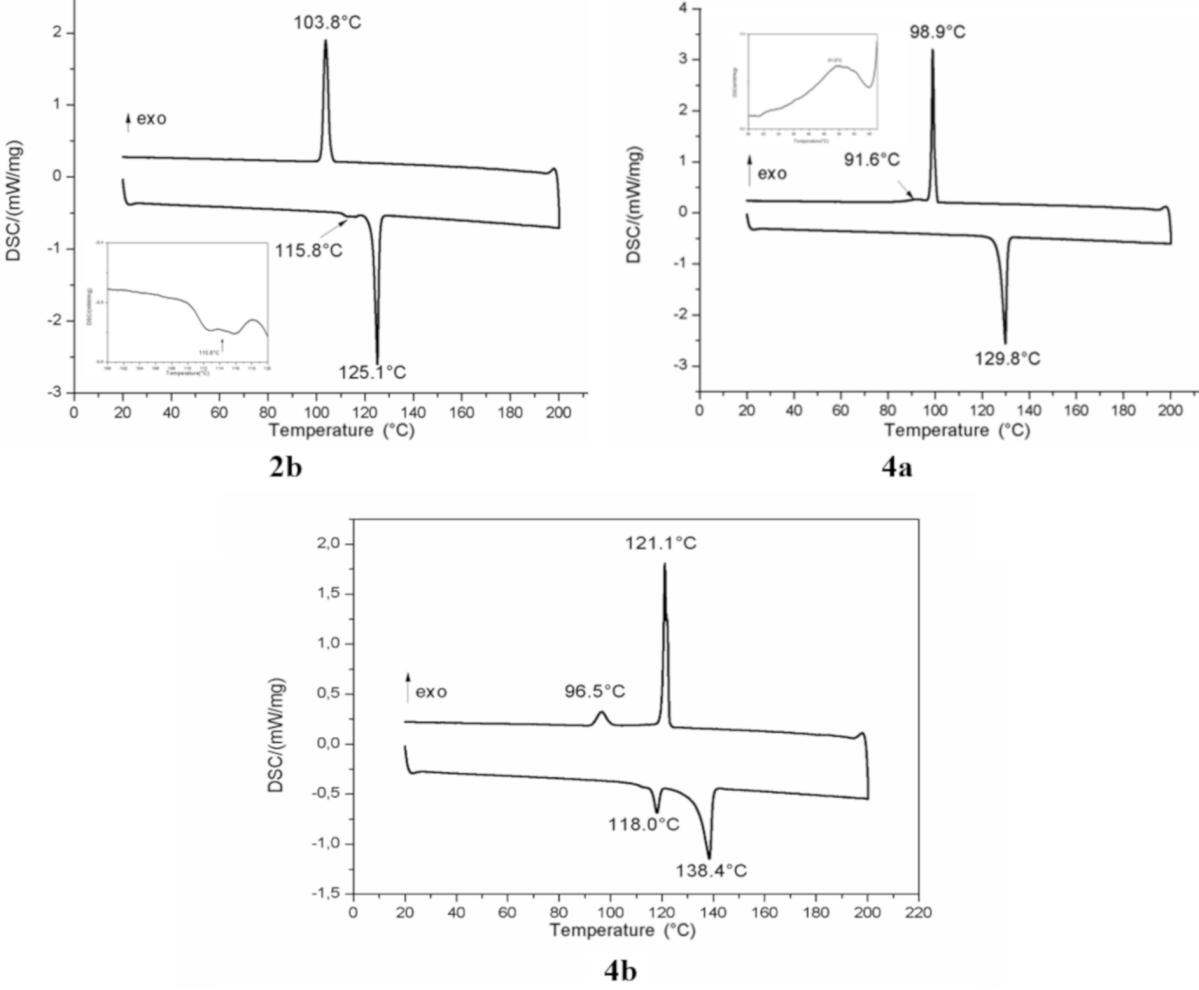
DSC thermograms of fluorinated compounds **2b**, **4a** and **4b** recorded at 5 °C/mn at heating (down traces) and cooling (top traces) cycles.

DSC thermograms of fluorinated compounds **2b**, **4a** and **4b** are shown in Figure 1. The thermogram of derivative **2b** presents only one peak between the crystalline and the isotropic phase in cooling. However, it exhibits two peaks in heating which indicates the presence of monotropic intermediate phase between *T* = 115.8 °C and *T* = 125.1 °C [30]. As it can be seen in the thermogram of compound **4a**, the monotropic liquid crystal mesophase is observed in cooling between *T* = 91.6 °C and 98.9 °C.

Concerning compound **4b**, the thermogram shows an enantiotropic intermediate phase between 118.0 °C and 138.4 °C in heating and between 96.5 °C and 121.1 °C in cooling, indicating a remarkable stabilization in a temperature range of 20 °C.

#### Polarized optical microscopy (POM)

**Figure 2:**
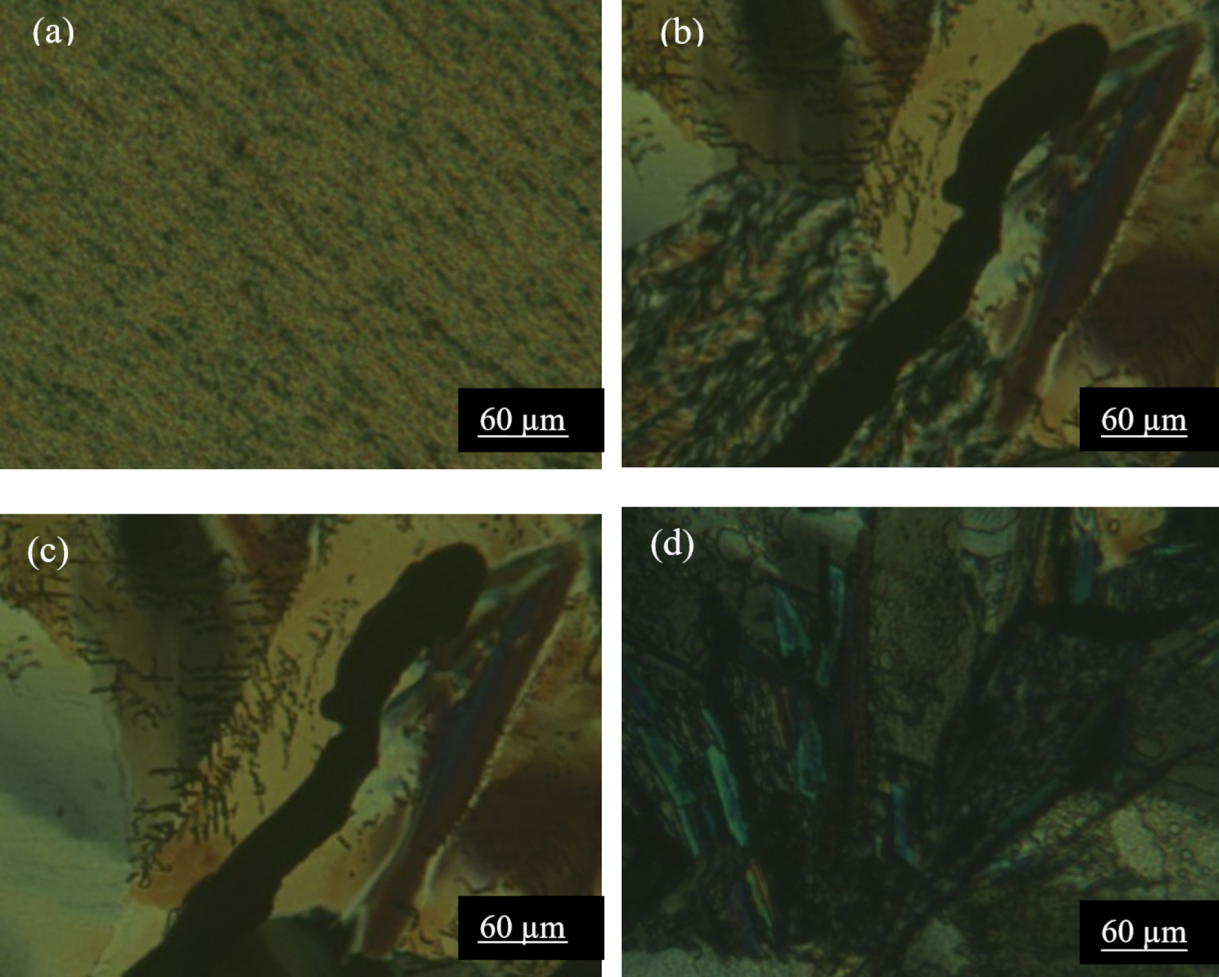
Optical texture (×10) of liquid crystal phase for fluorinated compounds, (a): SmA phase observed in heating at 120 °C for compound **2b**; (b): Coexistence between cristalline phase and liquid crystal phase observed in cooling at 91 °C for compound **4a**; (c): Mosaic SmB phase observed at 96 °C for compound **4a** and (d): SmB phase observed in heating cycle at 126 °C for compound **4b**.

In order to achieve a further illustration of the liquid crystal behavior, POM observations were realized in cooling and heating cycles for compounds **2b**, **4a** and **4b** (Figure 2). POM technique has illustrated smectic phases for all compounds. Identification of the phase textures was accomplished by comparing with those reported in literature [24].

The texture of **2b** (Figure 2a) seems to be a variant of focal conic texture with unusually narrow ellipses of a SmA phase. Compound **4a** presents a mosaic SmB phase where the molecules are organized in a hexagonal network as shown in Figure 2b. The low average of enthalpy value (1.89 J/g) given in Table 2 is due to the first order phase transition marked by the coexistence of the crystalline phase and the liquid crystal phase at least 5 °C as it is shown in Figure 2c. Figure 2d shows the POM observation for compound **4b** in heating cycles at 126 °C. The texture in cooling is very similar to that in heating; this indicates a remarkable thermodynamic stability of the compound. Based on the texture in Figure 2d, we can note that the mesophase is hexagonal SmB with strongly double refracting lancets and regions [31].

Under crossed polarizes, compound **2d** is not capable to induce any liquid crystalline behaviour, they present only crystal phases. Gallardo et al. investigated on the liquid crystalline behaviour of some bis(phenyl-1,3,4-oxadiazolyl)benzene derivatives with varied number and length of terminal alkoxy chains [32]. The authors established that the mesogenicity is strongly enhanced in materials with four long terminal alkoxy substituents, compared to two-chain and shorter-chain homologues. Taking into account these observations, the inability of **2d** to exhibit mesophases becomes axiomatic, since this compound is devoid of any terminal alkyl chain.

#### X-ray patterns analysis

**Figure 3:**
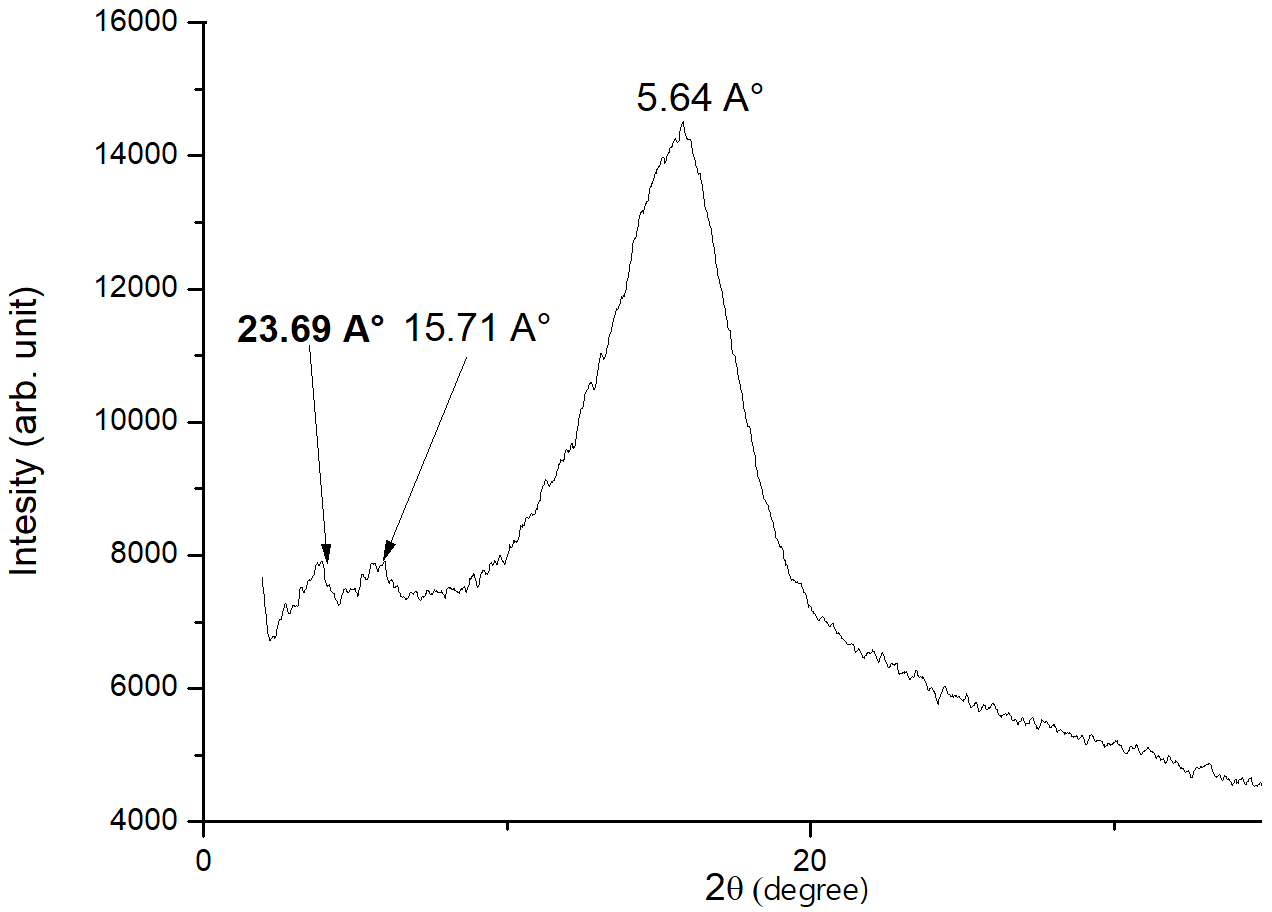
Typical diffractogram observed for compound **2b** at 398 K.

**Figure 4:**
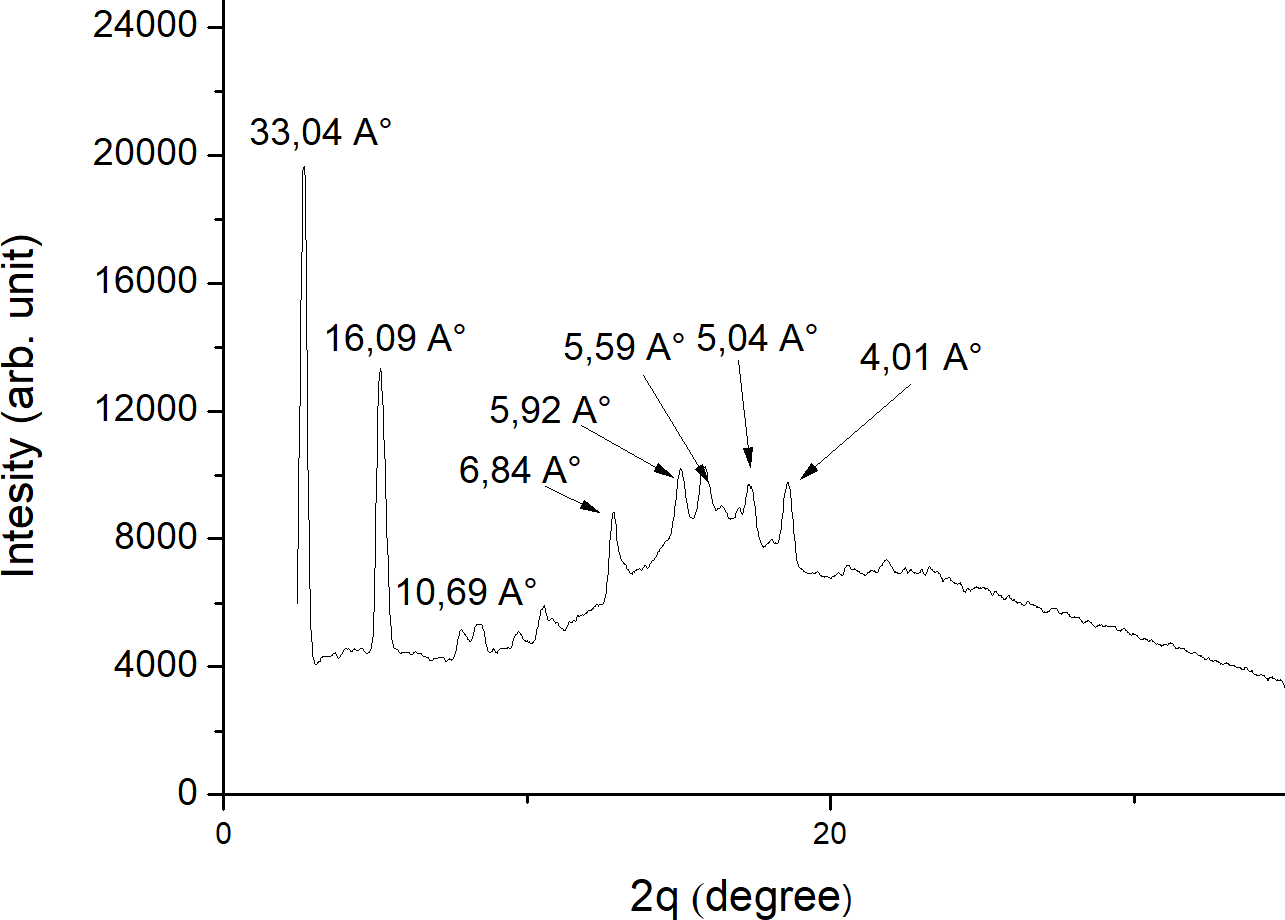
Typical diffractogram observed for compound **4a** at 411 K.

In order to correlate the obtained results from POM and DSC, we have investigated the X-ray diffraction at the mesophases in cooling and heating cycle of **2b**, **4a** and **4b**. Figure 3 illustrates typical diffraction spectra for compound **2b**. A typical X-ray pattern recorded for the SmA phase is obtained, it showed two small reflections at 23.69 Å and 15.71 Å at low angle region, and a broad reflection at 5.64 Å. These features are characteristic of SmA phase and are close to data described in the literature [33]. Figure 4 shows a typical X-ray pattern for the hexagonal SmB of **4a** (anyway, we must emphasize that both compounds **4a** and **4b** exhibit an identical X-ray pattern). As we can observe, three peaks of diffraction are recorded. The diffraction pattern shows oriented reflections in the small angle region. Bragg peaks at 33.04 Å, and approximately its second- and third order multiples at *d* = 16.09 Å and 10.69 Å, indicate a highly condensed layered structure [34].

However, in the high 2θ-region of Figure 4, slightly different values of the *d*-spacings calculated from these peaks suggest a weakly distorted hexagonal lattice [34].

### Structure–conformation relationships

#### Molecular dipole moment

Calculated electric dipole moments of compounds **2** and **4** are reported in Table 3.

**Table 3:** Calculated dipole moments of **2** and **4**.

Compound	Dipole moment (D)			

	Components			Total

	X	Y	Z	

**2a**	0.08	1.54	-4.48	4.74
**2b**	−0.04	1.95	−4.30	4.72
**2c**	−1.20	−3.34	0.71	3.61
**2d**	−0.93	2.59	0.38	2.78
**4a**	−1.25	4.35	−0.75	4.58
**4b**	1.40	5.13	−0.67	5.63
**4c**	−1.51	0.85	−0.22	1.74
**4d**	−1.35	0.77	−0.67	1.70

**Figure 5:**
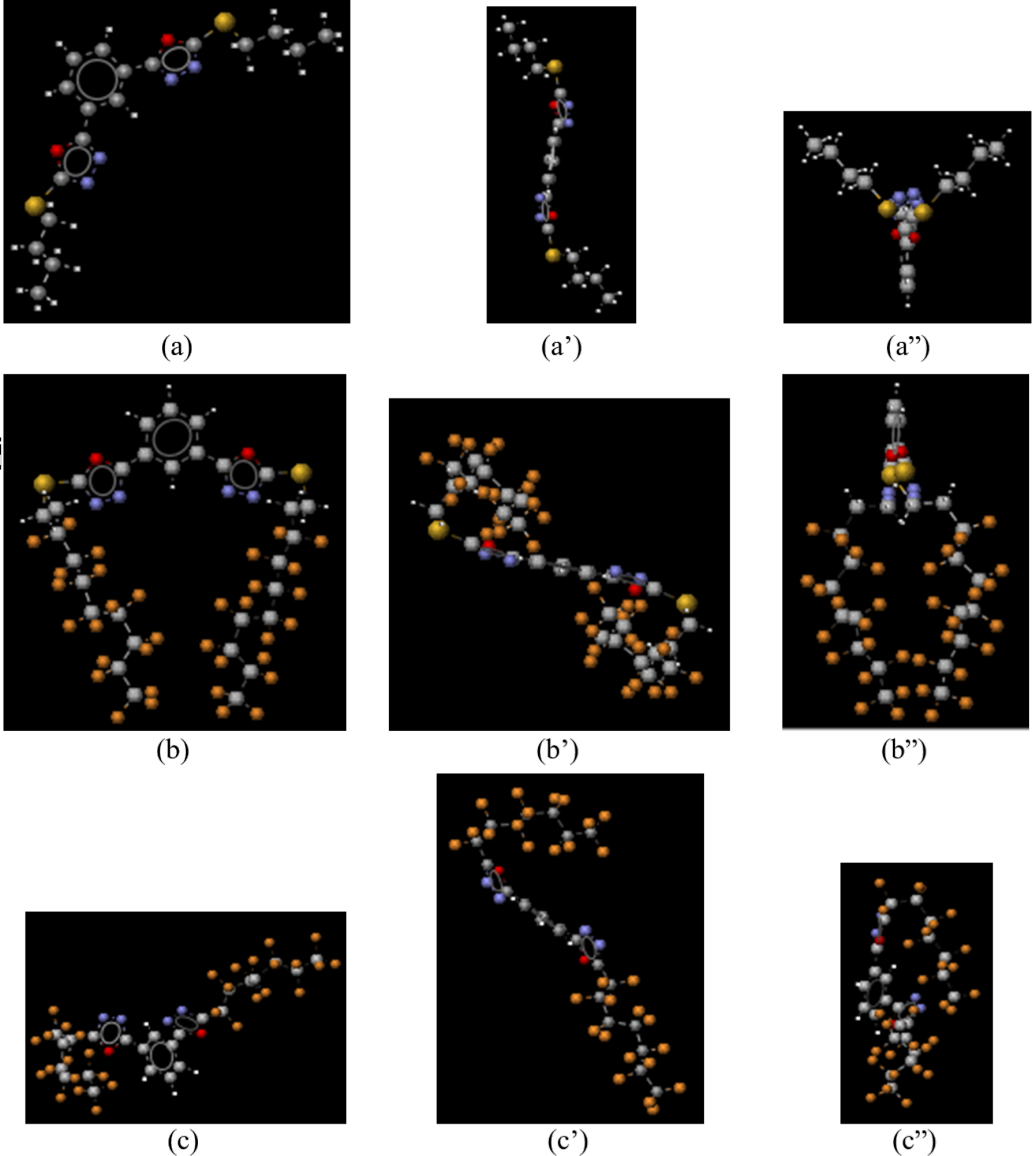
Conformer of lowest energy of compounds: **4c**, conformation A, (a) front view, (a’) top view, (a”) side view; **4b**, conformation B, (b) front view, (b’) top view, (b”) side view; **2b**, conformation C, (c) front view, (c’) top view, (c”) side view. Carbon atoms are shown in gray, hydrogen atoms in white, sulfur atoms in yellow, nitrogen atoms in blue, oxygen atoms in red and fluorine atoms in orange.

The prepared compounds perform three kinds of conformations, A, B and C. The hydrocarbon derivatives exhibit conformation A (Figure 5a–a”), whereas the fluorocarbon analogues adopt, depending on whether they carry linking segment C_2_H_4_S or not, the conformation B (Figure 5b–b”) or C (Figure 5c–c”).

**Figure 6:**
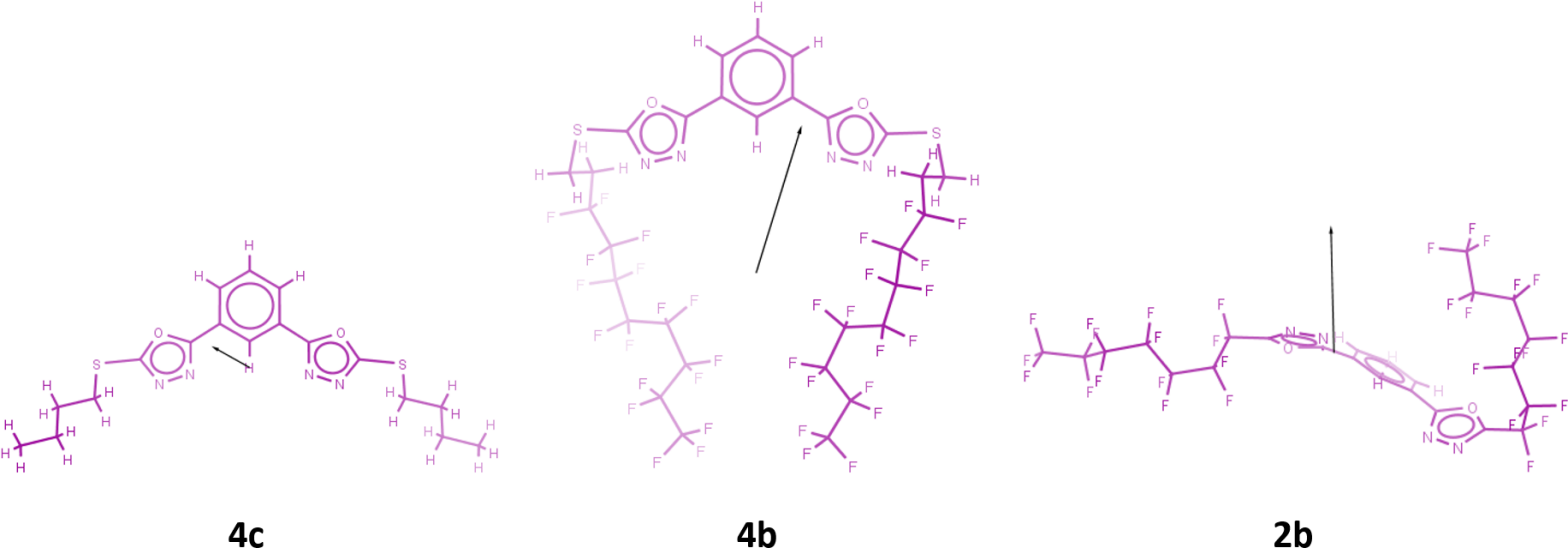
Vector of dipole moment of compounds **4c**, **4b** and **2b**.

In Figure 6 the vectors of the dipole moments of compounds **4c**, **4b** and **2b** are shown.

**Figure 7:**
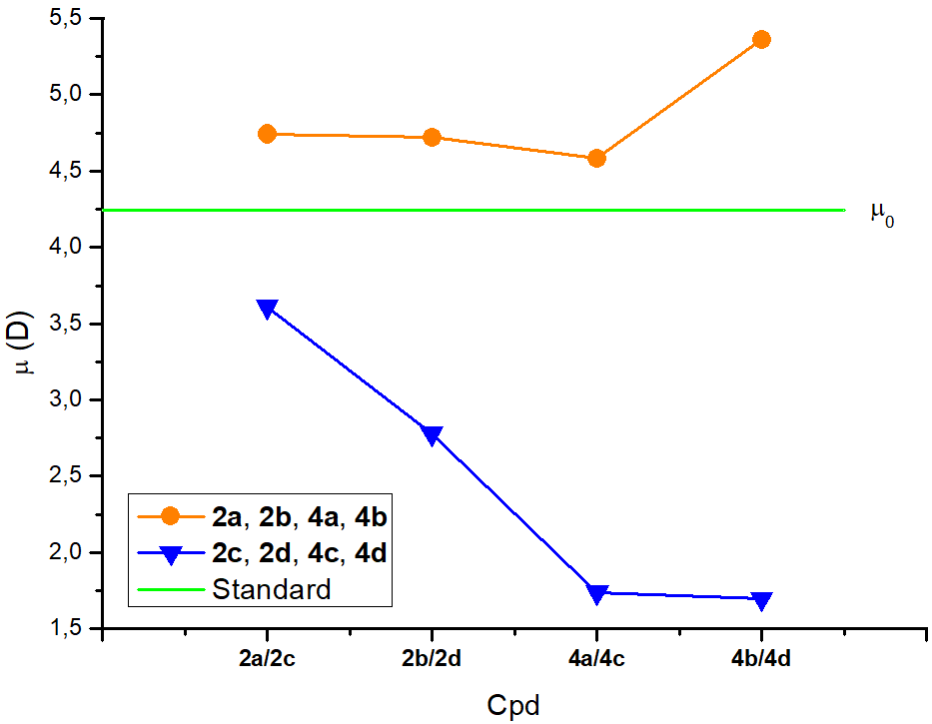
Plot of molecular dipole moments. Orange, fluorocarbon compounds; blue, hydrocarbon compounds; green (horizontal line), dipole moment (µ_0_) of RC.

In Figure 7 we plotted the calculated dipole moments. To quantify the effect of terminal chains, we calculated the dipole moment µ_0_ of 1,3-bis(1,3,4-oxadiazol-2-yl)benzene (RC) as it contains no terminal tails and can serve as standard.

The oxadiazole moieties are thought to be responsible for the majority of the dipole moment in the prepared compounds. Due to the electron-donating effect of alkyl groups in combination with the electron-accepting oxadiazole [35], the prepared hydrocarbon compounds are expected to perform dipole moment magnitudes higher than µ_0_. In the fluorinated counterparts, the electron-withdrawing effect of perfluoroalkyl groups would produce a reverse effect leading to a dipole lower than µ_0_. Figure 7 shows an inverse result: the orange plot corresponding to the fluorocarbon derivatives is entirely in the upper side compared to the horizontal green line (standard µ_0_), the blue plot of the hydrocarbon derivatives being in the bottom side.

On the other hand, we can note from Figure 5 that the conformation adopted by the hydrocarbon compounds is different from that of the fluorinated homologues, moreover these latter do not adopt the same conformation, according to whether they carry a sulfur atom or not.

Obviously, the inductive effects alone are inconsistent with the observed results. Hence, we considered the rigid-core (RC) and the terminal chains separately in order to identify a possible interaction between them.

#### Rigid-core

With three electronegative heteroatoms and only two carbons, the 1,3,4-oxadiazole core has a great electron deficient character. Nevertheless, when incorporated in the rigid-core, the two oxadiazole rings exhibit a slight difference in their electron deficiency.

We depicted in Table 4 the electric charge of heteroatoms in the obtained compounds, as well as those of RC. As shown in Table 4, with the exception of O13, all the other heteroatoms have almost the same electrical charge. The mean value of electric charge for O13 is −0.27. Hence, O13 is highly charged relative to the other heteroatoms, suggesting that Oxd 1 is more polar than Oxd 2.

**Table 4:** Electric charge of heteroatoms in compounds **2** and **4**.

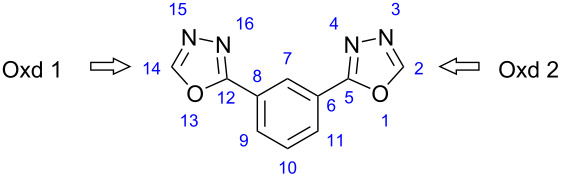						

Cpd.	O13	N15	N16	O1	N4	N3

**2a**	−0.25	−0.14	−0.10	−0.16	−0.14	−0.13
**2b**	−0.25	−0.14	−0.10	−0.16	−0.14	−0.13
**4a**	−0.28	−0.14	−0.13	−0.15	−0.13	−0.12
**4b**	−0.28	−0.14	−0.13	−0.15	−0.13	−0.12
**2c**	−0.29	−0.17	−0.12	−0.18	−0.15	−0.15
**2d**	−0.26	−0.16	−0.13	−0.18	−0.15	−0.15
**4c**	−0.28	−0.14	−0.13	−0.15	−0.13	−0.12
**4d**	−0.28	−0.14	−0.13	−0.15	−0.13	−0.12
mean value	−0.27	−0.15	−0.12	−0.16	−0.14	−0.13
RC	−0.28	−0.16	−0.11	−0.18	−0.15	−0.15

Such a difference in polarity was corroborated by the direction of the dipole moment of RC (Table 5). Therefore, it is not surprising to find the molecular electron-deficient center so far from the axis of the molecule in the prepared compounds.

**Table 5:** Dipole moment of RC.

		µ (D)			
Compound		X	Y	Z	Total

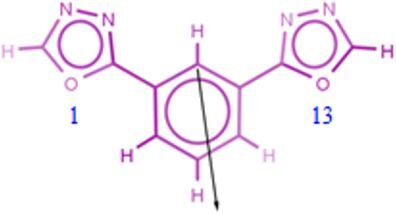	RC	0.59	−4.20	−0.01	4.24

#### Terminal chains

The intrinsic difference between fluorocarbon and hydrocarbon chains has to be taken into account to elucidate the conformational arrangements of the prepared compounds.

Because of electrostatic repulsions of fluorine atoms in the relative 1,3-positions in the crystalline state, the perfluorocarbon chain adopt a helical conformation of the carbon backbone [36]. The cylinder-like structure of the segment (CF_2_)*_m_* resembles a stiff rod in which the carbon skeleton is covered by fluorine atoms. In the hydrocarbon counterpart the segment (CH_2_)*_n_* adopts the typical in-plane zigzag conformation [36].

Fluorine is the most electronegative element of the periodic table. This high electronegativity confers to C–F bond a large dipole moment of 1.39 D while that of C–H bond is only 0.40 D [37]. Owing to the all-*trans* conformation, the local dipole moments C^δ−^–H^δ+^ of hydrocarbon chain are mutually neutralized. In (CF_2_)*_m_* segment, Hasegawa et al. noted that the local dipoles C^δ+^–F^δ−^ cannot be cancelled out and the surface of fluorocarbon chain remains polar [38].

#### Interaction rigid core-terminal chains

The typical model for aromatic electron donor–acceptor (D–A) interactions was established by Hunter and Sanders in 1990 [39]. According to this model, benzene and hexafluorobenzene form a complex where benzene is the donor (electron-rich) and hexafluorobenzene is the acceptor (electron-deficient). Experimental evidence for this complex was first reported in 1960 [40]. The electron D–A concept may be regarded as Lewis base–Lewis acid type or charge-transfer.

Based upon the above considerations, we could attribute the close proximity of fluorinated chains in conformation B to a throw space electron D–A intramolecular interaction between the perfluoroalkyl chains (electron-rich moieties) and the electron-deficient center of the molecule. The two fluorocarbon chains are symmetrically arranged with respect to the origin of the vector of dipole moment (Figure 6, compound **4b**). In compound **4b**, which adopts the conformation B (Figure 5b–b”), the fluorinated terminal chains resemble a twin [41]. The molecule is linear in the meaning that the rigid-core is in one side and the two arms in the other. Thus arranged, the molecule is polar and performs liquid crystal phases.

The presence of the linking group C_2_H_4_S in compounds **4a** and **4b** has the desired effect of increasing conformational flexibility, bringing the two fluoroalkyl chains closer. As a result, **4a** and **4b** adopt the conformation B (Figure 5b–b”). However, although the non-fluorinated analogues **4c** and **4d** also carry the segment C_2_H_4_S, an electron D–A interaction was not observed in these compounds, obviously because of the fundamental difference between fluoro- and hydrocarbon chains mentioned above. The conformation A adopted by these compounds (Figure 5a–a”) lowers the dipole moments and thereby precludes mesophases formation. In the same way, we can rationalize the absence of mesomorphism in compound **2c** which is not only non-fluorinated, but also devoid of the segment C_2_H_4_S.

On the other hand, direct linkage of perfluoroalkyl groups to the rigid-core makes the system more rigid. As shown in Figure 5c–c”, the conformation C adopted by compound **2b** is quite different from conformation B described above. While R_F_2 (R_F_ linked to Oxd 2) folds to interact with the molecular electron-deficient center according to an electron D–A interaction, R_F_1 (R_F_ linked to Oxd 1) remains straight. The dipole moment of compound **2b** (Figure 6) originates close Oxd 1. With the presumption that the molecular electron-deficient center is near the origin of the vector, we can attribute the absence of folding on the Oxd 1 side to the huge sprain that R_F_1 must undergo to interact with a center that is so close to it.

## Conclusion

In this paper we have described the preparation and characterization of two new series of 2,2'-(1,3-phenylene)bis(1,3,4-oxadiazole) derivatives bearing different hydro- and fluorocarbonated chains. Structures of the obtained compounds were established by usual spectroscopic techniques. DSC, POM and X-ray diffraction investigations evidence the existence of the liquid crystal mesophase in the perfluorinated derivatives whereas the hydrocarbonated counterparts just present a thermotropic character. The dipole moment-molecular conformation relationship was scrutinized in order to elucidate the role of the molecular conformation on the dipole moment magnitude. Since some of the studied compounds show mesomorphic properties, it is important to lead on a deep study in order to investigate other parameters such as response time, viscosity and dielectric anisotropy.

## Supporting Information

File 1 Experimental procedures, characterization data, NMR and FTIR spectra for the reported compounds and DSC thermograms for **2a** and **4d**.
