# Supplementary material for: Synthesis, liquid crystalline behaviour and structure–property relationships of 1,3-bis(5-substituted-1,3,4-oxadiazol-2-yl)benzenes
Source: Beilstein J Org Chem. 2020 Jan 31;16:149–58. doi: 10.3762/bjoc.16.17 (PMC7006484; doi:10.3762/bjoc.16.17)
Supplement: File 1 — Experimental procedures, characterization data, NMR and FTIR spectra for the reported compounds and DSC thermograms for 2a and 4d. [file Beilstein_J_Org_Chem-16-149-s001.pdf]

BEILSTEIN JOURNAL OF ORGANIC CHEMISTRY

Supporting Information

for

Synthesis, liquid crystalline behaviour and structure–property relationships of 1,3-bis(5-substituted-1,3,4-oxadiazol-2-yl)benzenes

Afef Mabrouki, Malek Fouzai, Armand Soldera, Abdelkader Kriaa and Ahmed Hedhli

Beilstein J. Org. Chem. **2020,** *16,* 149–158. doi:10.3762/bjoc.16.17

Experimental procedures, characterization data, NMR and FTIR spectra for the reported compounds and DSC thermograms for 2a and 4d

License and Terms: This is a supporting information file under the terms of the Creative Commons Attribution License (https://creativecommons.org/licenses/by/4.0). Please note that the reuse, redistribution and reproduction in particular requires that the authors and source are credited.
The license is subject to the *Beilstein Journal of Organic Chemistry* terms and conditions: (https://www.beilstein-journals.org/bjoc)

Contents

1. Experimental part S2–S5

2. NMR and IR spectra S6–S21

3. DSC thermograms for compounds **2a** and **4d** S22

Experimental part

Materials and analytical measurements

Commercially available reagents were all purchased from ACROS organics. Perfluoroheptanoic acid and perfluorohexanoic acid were of 96%, perfluoroethyl iodide reagents were of 99%, benzoic acid and decanoic acid were of 99%, bromododecane was of 99%, and carbon disulfide was of 99.9%. All other chemicals were of 98% and solvents were of analytical grade. The ^{1}H, ^{13}C and ^{19}F NMR spectra were recorded on a Bruker AC 300 at 300, 75 and 282 MHz, respectively. TMS was used as standard reference for ^{1}H NMR and ^{13}C NMR spectra and $CFCl_3$ for ^{19}F NMR. The IR spectra were recorded on a Bruker IFS 66V/S. High-resolution mass spectral analysis (HRMS) was performed on AMD-604 spectrometer. Melting points were determined in capillaries and they are uncorrected. Differential scanning calorimetry (DSC) thermograms, are obtained in heating and cooling cycle. The sample is heated and cooled with a scan rate of 5 $K \cdot min^{-1}$ and held at its isotropic phase for two minutes to attain the thermal stability. Transition temperatures were checked and type of mesophase identified for the samples using a standard polarized-optical microscope POM (Olympus BX51) equipped with digital CCD camera (Sony). The LC textures are processed, analyzed and stored with aid of imaging software (Archimed). To set the temperature we put the cell in an oven. The temperature was controlled within ±1°C/min. The X-ray patterns were collected at University of Sherbrooke, Qc. Canada, with a Bruker AXS Nanostar system equipped with a Microfocus Copper Anode at 45 kV / 0.65 mA, MONTAL OPTICS and a VANTEC 2000 2D detector. The detector to sample distance was calibrated with a Silver Behenate standard at 67.70 cm. The diffracted intensities were integrated from 0.15 to 5.2 deg. 2-theta. The collection exposure time was 300 seconds per sample.

Prediction of dipole moments and lowest conformation energies were accomplished by using the application *Instant J.Chem* 18.28.0; ChemAxon (http://www.chemaxon.com).

Synthesis of oxadiazole derivatives ***2a–d****: General procedure*

A mixture of benzene-1,3-dicarbohydrazide (0.97 g, 1 mmol) and 2 mmol of carboxylic acid in 10 ml of phosphorus oxychloride was refluxed for 12 h. The reaction mixture was slowly poured over crushed ice and kept overnight. The resulting solid was washed with aqueous $NaHCO_3$ and then with water and recrystallized from dimethyl formamide.

1,3-Bis(5-perfluorohexyl-1,3,4-oxadiazol-2-yl)benzene (**2a**): Pink solid; yield = 65%; m.p.: 106°C; IR (cm^{-1}): $\nu_{C=N}$: 1550, $\nu_{C=C}$: 1500, $\nu_{C\text{-}O:}$ 1144, $\nu_{C\text{-}F:}$ 1145; ^{1}H NMR (300 MHz, $CDCl_3$) δ (ppm): 7.80-8.81 (4H, $C_6\underline{H}_4$);^{13}C NMR (75 MHz, $CDCl_3$) δ (ppm): 165.72 (1s, 2C, N=$\underline{C}$-C_6H_4-$\underline{C}$=N), 155.40 (t, 2C, 2 N=$\underline{C}$-CF_2,$^{2}J_{CF}$ =23.00 Hz), 131.67, 130.72, 123.73, 119.00 (4s, 6C, $\underline{C}_6H_4$); ^{19}F NMR (282 MHz, $CDCl_3$) *δ* (ppm): -80.80 (m, 6F, $2CF_3$), -112.56 (m, 4F, $2CF_{2\alpha}$), -121.53 (m, 4F, $2CF_{2\beta}$), -121.83 (m, 4F, $2CF_{2\gamma}$),-122.69 (m, 4F, $2CF_{2\delta}$), -126.08 (m, 4F, $2CF_{2\omega}$); HRMS (EI): calculated for $C_{22}H_4F_{26}N_4O_2$ 849.9919, found 849.9920.

1,3-Bis(5-perfluoroheptyl-1,3,4-oxadiazol-2-yl)benzene (**2b**): Pink solid; yield = 61%; m.p.: 120°C; IR (cm^{-1}): $\nu_{C=N}$: 1500, $\nu_{C=C}$: 1400, $\nu_{C\text{-}O:}$ 1145; ^{1}H NMR (300 MHz, $CDCl_3$) δ (ppm): 7.81-8.88 (4H, $C_6\underline{H}_4$); ^{13}C NMR (75 MHz, $CDCl_3$) δ (ppm): 165.41 (1s, 2C, N=$\underline{C}$- C_6H_4-$\underline{C}$=N), 154.9 (t, 2C, 2N=$\underline{C}$-$CF_{2,}$$^{2}J_{CF}$ = 23.30 Hz), 130.86, 129.56, 124.65, 120.24 (4s, 6C, $\underline{C}_6H_4$); ^{19}F NMR (282 MHz, $CDCl_3$) *δ* (ppm): -80.74 (m, 6F, $2CF_3$), -112.54 (m, 4F, $2CF_{2\alpha}$), -121.37 (m, 4F, $2CF_{2\beta}$) -121.84 (m, 8F, $4CF_{2\gamma}$), -122.64 (m, 4F, $2CF_{2\delta}$), -126.09 (m, 4F, $2CF_{2\omega}$); HRMS (EI): calculated for $C_{24}H_4F_{30}N_4O_2$ 949.9855, found 949.9855.

1,3-Bis(5-nonyl-1,3,4-oxadiazol-2-yl)benzene (**2c**): White solid; yield = 75%; m.p.: 83°C; IR (cm^{-1}) $\nu_{(C–H)}$: 2918, $\nu_{C=N}$: 1547, $\nu_{C=C}$: 1479, $\nu_{C\text{-}O}$: 1250; ^{1}H NMR (300 MHz, $CDCl_3$) δ (ppm) 7.71-8.55 (4H, $C_6\underline{H}_4$), 3.33 (t, 4H, 2N=C-C$\underline{H}_2$$CH_2$, $^{3}J_{H\text{-}H}$ = 6.43 Hz), 1,22-1.88 (m, 28H, 2N=C-CH_2-(C$\underline{H}_2$)$_7$-CH_3), 0.80 (t, 6H, C$\underline{H}_3$$CH_2$, $^{3}J_{H\text{-}H}$ = 6.81 Hz); ^{13}C NMR (75 MHz, $CDCl_3$) δ (ppm): 165.53 (1s, 2C, N=$\underline{C}$-C_6H_4-$\underline{C}$=N), 163.75 (1s, 2C, 2N=$\underline{C}$-R), 129.87, 129.40, 125.11, 124.95 (4s, 6C, $\underline{C}_6H_4$), 31.82, 29.68, 29.35, 29.21, 29.11, 29.04, 29.59, 25.49 (8s, 16C, 2N=C-($\underline{C}H_2$)$_8$-CH_3), 14.09 (1s, 2C, 2$\underline{C}H_3$); HRMS (EI): calculated for $C_{28}H_{42}N_4O_2$ 466.3307, found 466.3311.

1,3-Bis(5-phenyl-1,3,4-oxadiazol-2-yl)benzene (**2d**): White solid; yield = 72%; m.p.: 74°C; IR (cm^{-1})$\nu_{C=N:}$1547, $\nu_{C=C}$: 1479, $\nu_{C\text{-}O:}$ 1250; ^{1}H NMR (300 MHz, $CDCl_3$) δ (ppm): 7.31-8.91 (14H, $C_6\underline{H}_4$, H_{2Ph}); ^{13}C NMR (75 MHz, $CDCl_3$) δ (ppm): 165.65 (1s, 2C, N=$\underline{C}$-C_6H_4-$\underline{C}$=N),164.22 (1s, 2C, 2$\underline{C}$=N-Ph), 132.34, 131.22, 129.41, 128.75, 127.63, 127.34, 126.4, 124.83 (8s, 18C, $\underline{C}_6H_4$, C_{2Ph}); HRMS (EI): calculated for $C_{22}H_{14}N_4O_2$ 366.1116, found 366.1114.

Preparation of bis-1,3-(5-thio-1,3,4-oxadiazol-2-yl)benzene 3

To a solution of potassium hydroxide (0.56 g, 10 mmol) in absolute ethanol (50 ml), isophthalic dihydrazide **1** (0.97 g, 5 mmol) was added with stirring. Carbon disulfide (0.83 g, 11 mmol) was added to the reaction mixture, which led to a pale yellow precipitate formation. The

reaction mixture was heated under reflux for overnight, during which time the mixture became clear. The solvent was removed under reduced pressure, and the residue was dissolved in cold water. Then it was acidified with glacial acetic acid and filtered off to give the crude product. The latter was recrystallized from DMF. Yellow solid, yield: 54%, m.p.: 218°C. IR (cm^{-1}): ν_{N-H} = 3387.1, ν_{C-H} = 3073, $\nu_{C=N}$ =1637.5, $\nu_{C=C}$ 1521.3, $\nu_{C=S}$ =1263.6, ν_{C-O-C} = 1160. ^{1}H NMR (300 MHz, DMSO) δ (ppm): 7.81-8.33 (4H, $C_6\underline{H}_4$), 3.55 (b, 1H, NN$\underline{H}$), ^{13}C NMR (75 MHz, DMSO) δ (ppm): 177.61 (1s, 2C, $\underline{C}$=S), 159.38, 162.26 (2s, 2C, N=$\underline{C}$-C_6H_4-$\underline{C}$=N), 123.05, 123.68, 129.15, 130.77 (4s, 6C, $\underline{C}_6H_4$).

*Synthesis of oxadiazole derivatives **4a–d**: General procedure*

To a solution of potassium hydroxide (0.28 g, 10 mmol) in absolute ethanol (50 ml), intermediate **3** (1.39 g, 5 mmol) was added under stirring. A solution of alkyl/fluoro alkyl ethyl halide (10 mmol) in ethanol (20 ml) was added. The reaction mixture was heated at 70 °C under reflux for overnight. After cooling, water (100 ml) was added and the resulting solid was recrystallized from ethanol/hexane.

1,3-Bis[(5-perfluorohexylethylsulfanyl)-1,3,4-oxadiazol-2-yl]benzene (**4a**): Pale yellow solid yield = 74%; m.p.: 128°C; IR(cm^{-1}): $\nu_{(C-H)}$:2920, $\nu_{C=N}$:1550, $\nu_{C=C}$:1471, ν_{C-O}: 1203; ^{1}H NMR (300 MHz, $CDCl_3$) δ (ppm): 7.55-8.71 (4H, $C_6\underline{H}_4$), 3.54 (t, 4H, 2SC$\underline{H}_2$-CH_2, $^3J_{H-H}$ = 6.77 Hz), 2.71 (m, 4H, 2SCH_2C$\underline{H}_2$$C_6F_{13}$); ^{13}C NMR (75 MHz, $CDCl_3$) δ (ppm): 165.54 (1s, 2C, N=$\underline{C}$-C_6H_4-$\underline{C}$=N), 162.42 (1s, 2C, 2N=$\underline{C}$-S$CH_2CH_2R_F$), 132.24, 131.33, 123.81, 121.00 (4s, 6C, $\underline{C}_6H_4$), 35.44 (t, 2C, S$CH_2$$\underline{C}H_2$$C_6F_{13}$, $^2J_{CF}$ = 21.96 Hz), 24.73 (1s, 2C, S$\underline{C}H_2CH_2CF_2$); ^{19}F NMR (282 MHz, $CDCl_3$) δ (ppm): -80.77 (m, 6F, 2CF_3), -114.32 (m, 4F, 2$CF_{2\alpha}$), -121.87 (m, 4F, 2$CF_{2\beta}$), -122.87 (m, 4F, 2$CF_{2\gamma}$), -123.33 (m, 4F, 2$CF_{2\delta}$), -126.14 (m, 4F, 2$CF_{2\omega}$); HRMS (EI): calculated for $C_{26}H_{12}F_{26}N_4O_2S_2$ 969.9986, found 969.9987.

1,3-Bis[(5-perfluorooctylethylsulfanyl)-1,3,4-oxadiazol-2-yl]benzene (**4b**): Pale yellow solid; yield = 66%; m.p.: 138°C; IR (cm^{-1}) $\nu_{(C-H)}$:2918, ν_{C-O}: 1203, $\nu_{C=N}$: 1552, $\nu_{C=C}$: 1471; ^{1}H NMR (300 MHz, $CDCl_3$) δ (ppm): 7.55-8.61 (4H, $C_6\underline{H}_4$), 3.58 (t, 4H, 2SC$\underline{H}_2$$CH_2$,$^3J_{HH}$ = 6.72 Hz), 2.84 (m, 4H, 2SCH_2C$\underline{H}_2$$C_8F_{17}$); ^{13}C NMR (75 MHz, $CDCl_3$) δ (ppm): 163.74 (1s, 2C, N=$\underline{C}$-C_6H_4-$\underline{C}$=N), 161.86 (1s, 2C, 2N=$\underline{C}$-S$CH_2CH_2R_F$), 131.7 6, 127.33, 124.55, 122.24 (4s, 6C, $\underline{C}_6H_4$), 35.7 (t, 2C, S$CH_2$$\underline{C}H_2$$CF_2$, $^2J_{CF}$ = 21.95 Hz), 24.71 (1s, 2C, S$\underline{C}H_2CH_2CF_2$); ^{19}F NMR (282 MHz, $CDCl_3$) *δ* (ppm): -80.72 (m, 6F, 2CF_3), -114.24 (m, 4F, 2$CF_{2\alpha}$), -121.62 (m, 4F, 2$CF_{2\beta}$), -121.87(m, 8F, 4$CF_{2\gamma}$), -122.67(m, 4F, 2$CF_{2\delta}$), -123.25 (m, 4F, 2$CF_{2\xi}$), -126.10 (m, 4F, 2$CF_{2\omega}$); HRMS (EI): calculated for $C_{30}H_{12}F_{34}N_4O_2S_2$ 1169.9858, found 1169.9862.

1,3-Bis[(5-butylsulfanyl)-1,3,4-oxadiazol-2-yl]benzene (**4c**): Pale yellow solid; yield = 70%; m.p.: 69°C; IR (cm^{-1}) $\nu_{(C\text{-}H)}$: 2954, $\nu_{C=N}$: 1553, $\nu_{C=C}$: 1465, $\nu_{C\text{-}O}$: 1181; ^{1}H NMR (300 MHz, $CDCl_3$) δ (ppm): 7.44-8.77 (4H, $C_6\underline{H}_4$), 3.33 (t, 4H, ,$2SC\underline{H}_2CH_2$, $^{3}J_{HH}$ = 6.83 Hz), 1.8 (m, 4H, $2SCH_2$ $C\underline{H}_2\text{-}CH_2$), 1.5 (m, 4H, $2CH_2C\underline{H}_2CH_3$), 1.10 (t ,6H, $2\underline{C}H_3$, $^{2}J_{HH}$ =7.32 Hz); ^{13}C NMR (75 MHz, $CDCl_3$) δ (ppm): 165.31, (1s, 2C, $N=\underline{C}\text{-}C_6H_4\text{-}\underline{C}=N$), 164.56 (1s, 2C, $2N=\underline{C}\text{-}R$), 129.93, 129.13, 124.74, 124.40 (4s, 6C, $\underline{C}_6H_4$), 32.36, 31.21, 21.74, (3s, 6C, $2(\underline{C}H_2)_3$), 13.50 (1s, 2C, 2 $\underline{C}H_3$); HRMS (EI): calculated for $C_{18}H_{22}N_4O_2S_2$ 390.1184, found 390.1183.

1,3-Bis[(5-dodecylsulfanyl)-1,3,4-oxadiazol-2-yl]benzene (**4d**): Pale yellow solid; yield = 72%; m.p.: 88°C; IR (cm^{-1}) $\nu_{(C\text{–}H)}$:2918, $\nu_{C\text{-}O:}$ 1176, $\nu_{C=N}$: 1550, $\nu_{C=C}$: 1471; ^{1}H NMR (300 MHz, $CDCl_3$) δ (ppm): 7.66-8.53 (4H, $C_6\underline{H}_4$), 3.3 (t, 4H, $2SC\underline{H}_2CH_2$, $^{3}J_{HH}$ = 6.70 Hz), 1.20-1.85 (m, 40H, $2SCH_2\text{-}(C\underline{H}_2)_{10}\text{-}CH_3$), 0.8 (t, 6H, $2C\underline{H}_3$, $^{3}J_{HH}$ = 7.41 Hz); ^{13}C NMR (75 MHz, $CDCl_3$) δ (ppm): 165.70, (1s, 2C, $N=\underline{C}\text{-}C_6H_4\text{-}\underline{C}=N$), 164.61 (1s, 2C, $2N=\underline{C}\text{-}R$), 130.09, 129.98, 124.86, 124.72 (4s, 6C, $\underline{C}_6H_4$), 34.03, 32.84, 32.72, 31.90, 29.69, 29.61, 29.54, 29.44, 29.40, 29.33, 29.32, (11s, 22C, $2(\underline{C}H_2)_{11}$), 14.11 (1s, 2C, $2\underline{C}H_3$); HRMS (EI): calculated for $C_{34}H_{54}N_4O_2S_2$ 614.3688, found 614.3679.

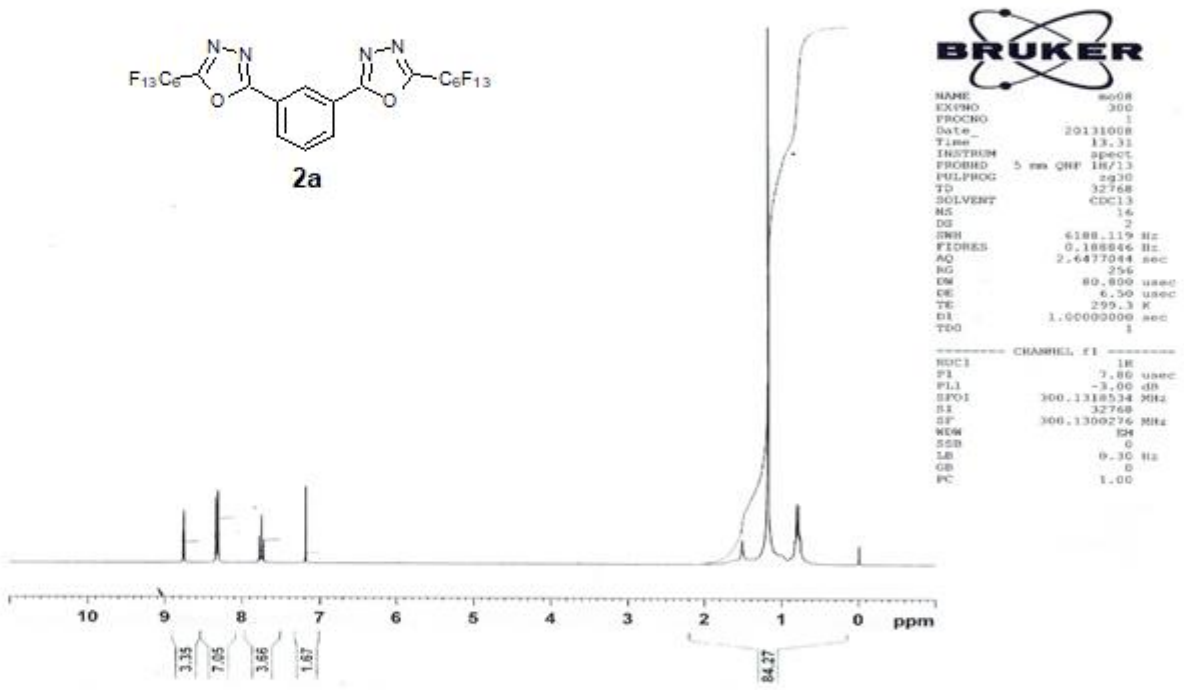

^{1}H NMR spectrum of compound **2a**.

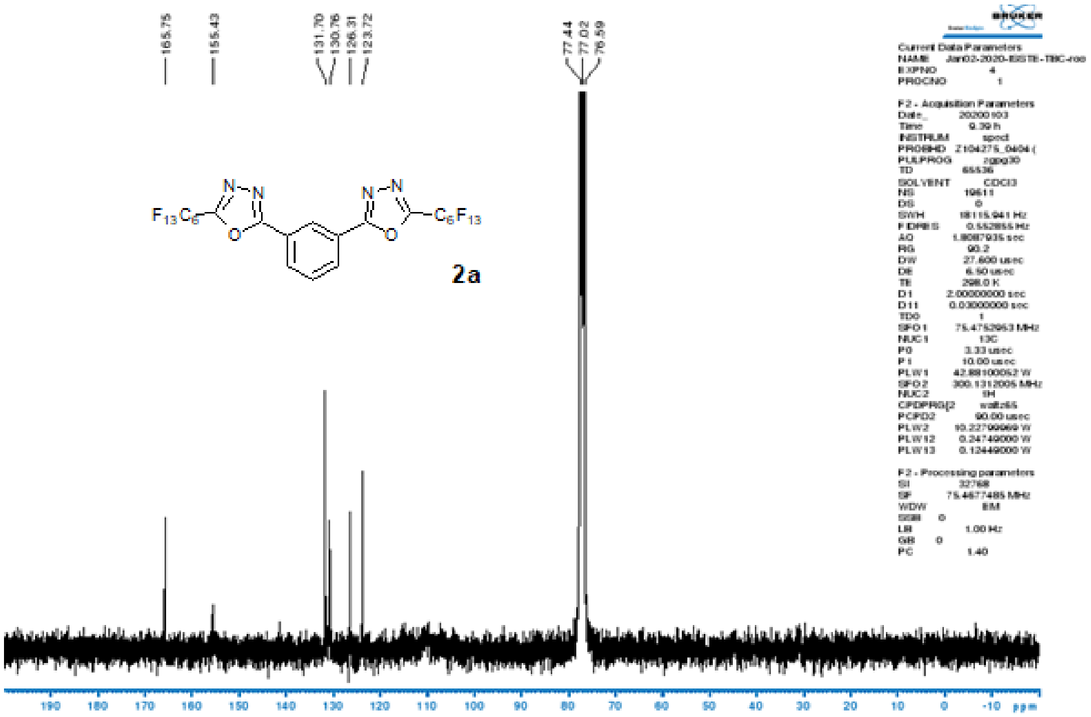

^{13}C NMR spectrum of compound **2a**.

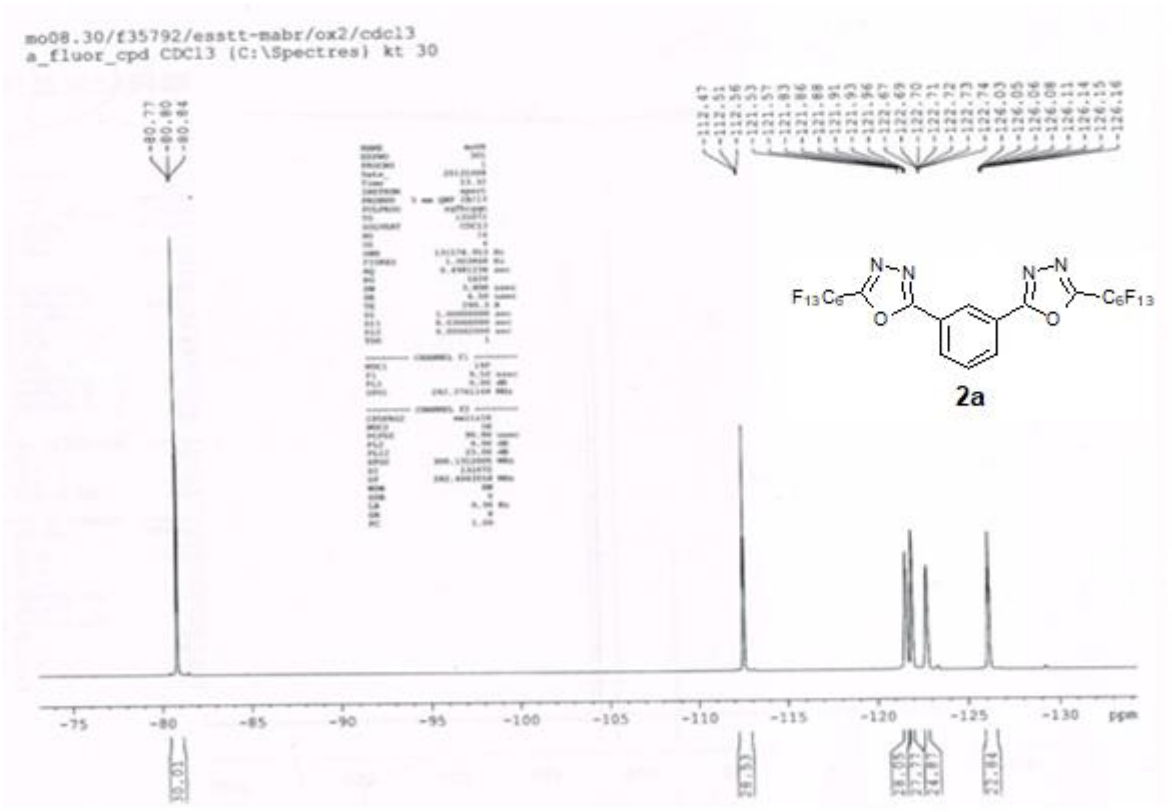

^{19}F NMR spectrum of compound **2a**.

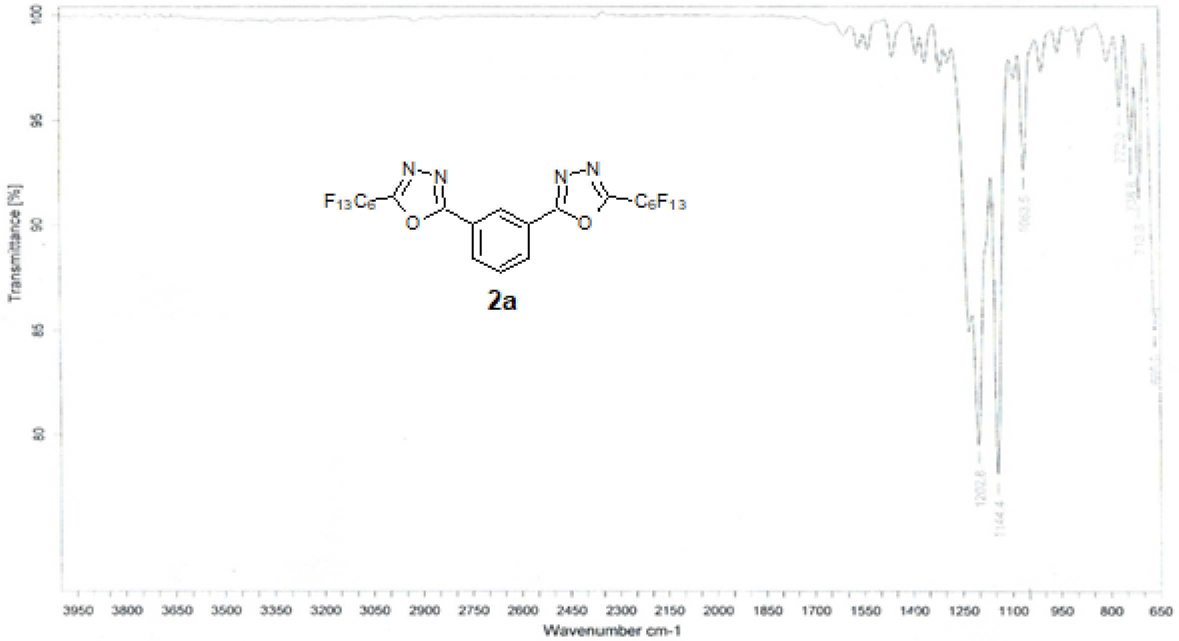

IR spectrum of compound **2a**.

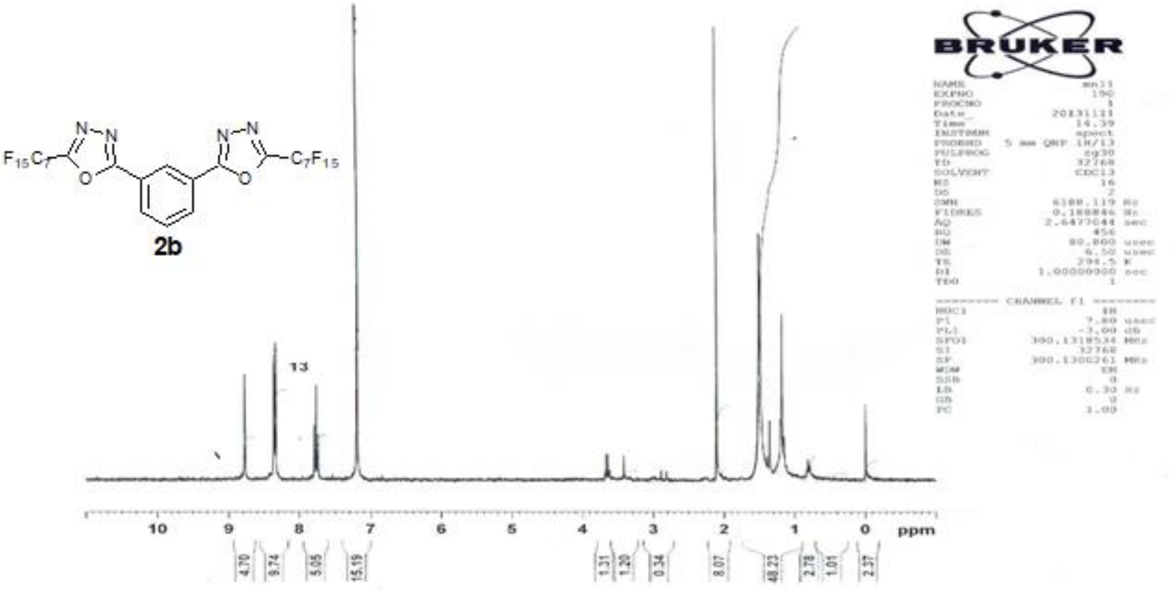

^{1}H NMR spectrum of compound **2b**.

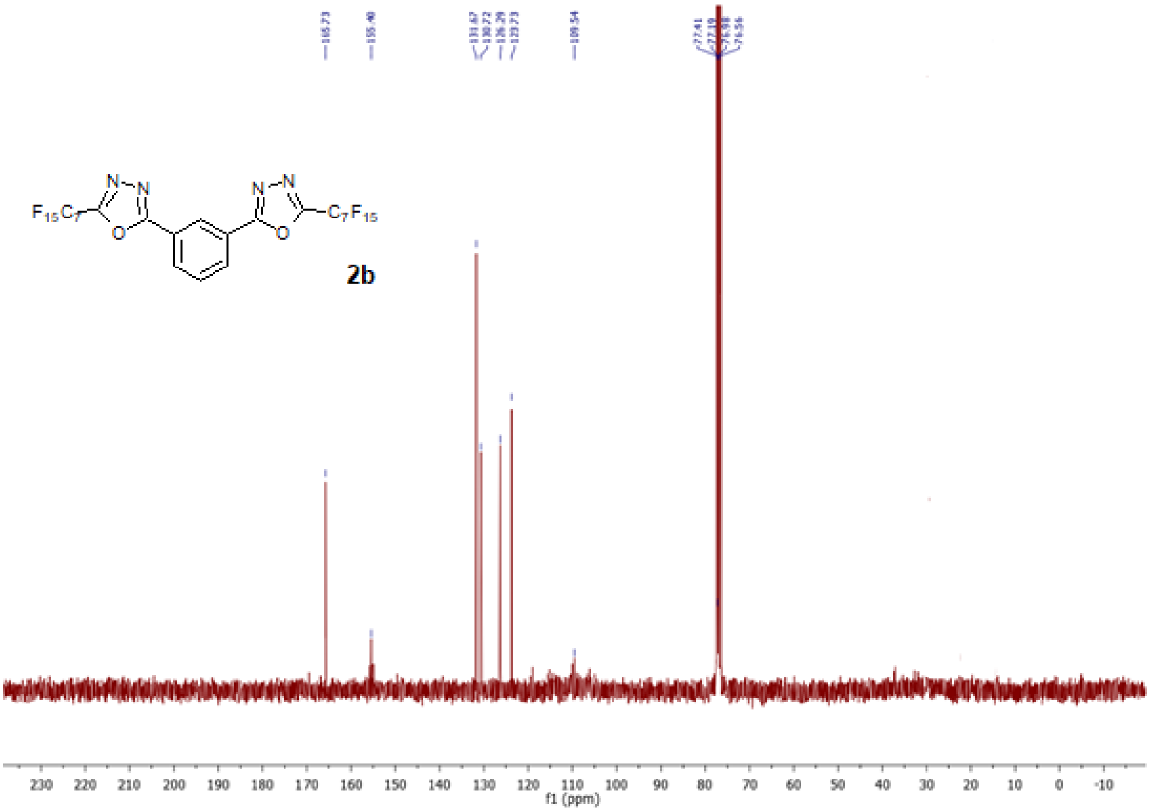

^{13}C NMR spectrum of compound **2b**.

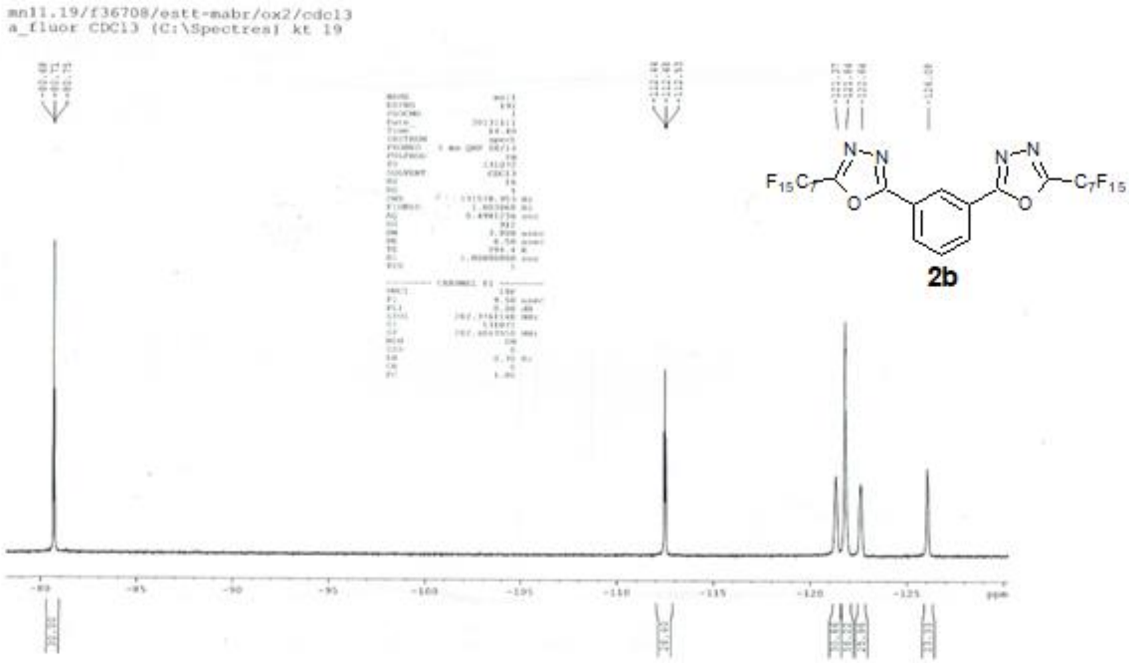

^{19}F NMR spectrum of compound **2b**.

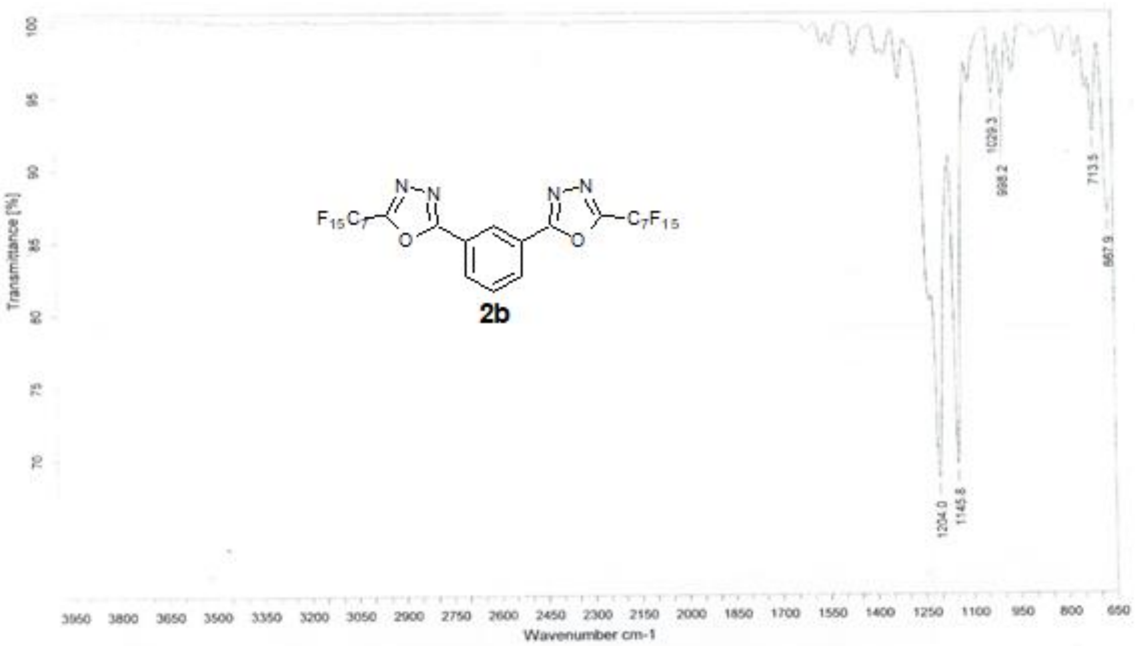

IR spectrum of compound **2b**.

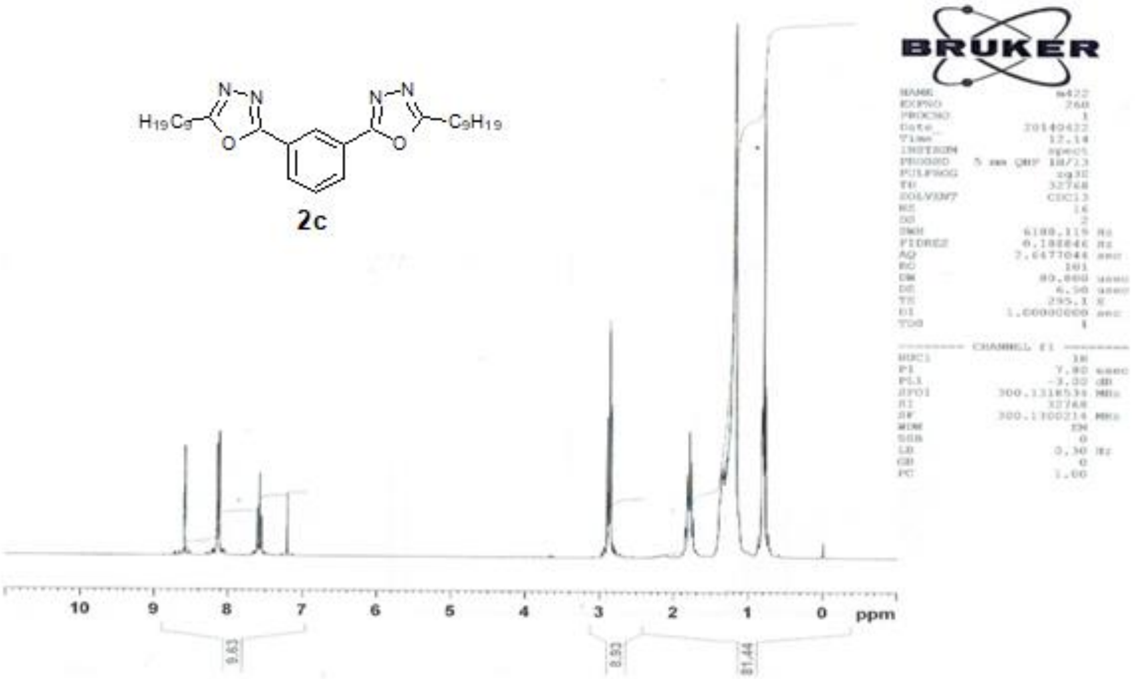

^{1}H NMR spectrum of compound **2c**.

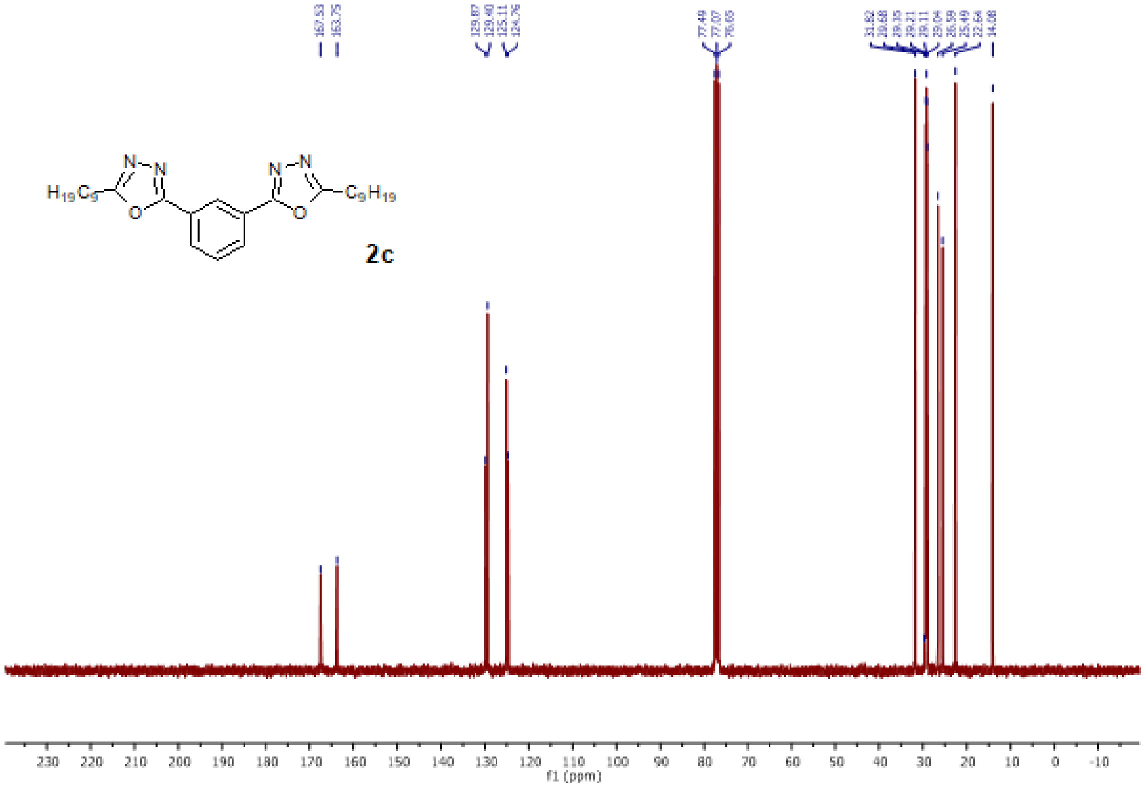

^{13}C NMR spectrum of compound **2c**.

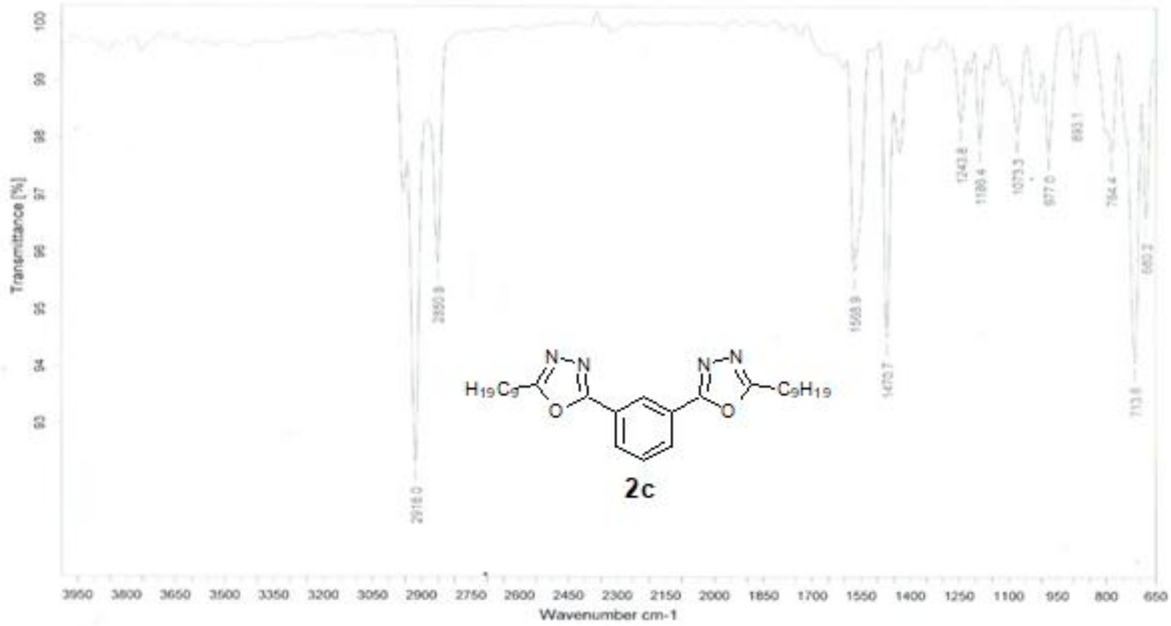

IR spectrum of compound **2c**.

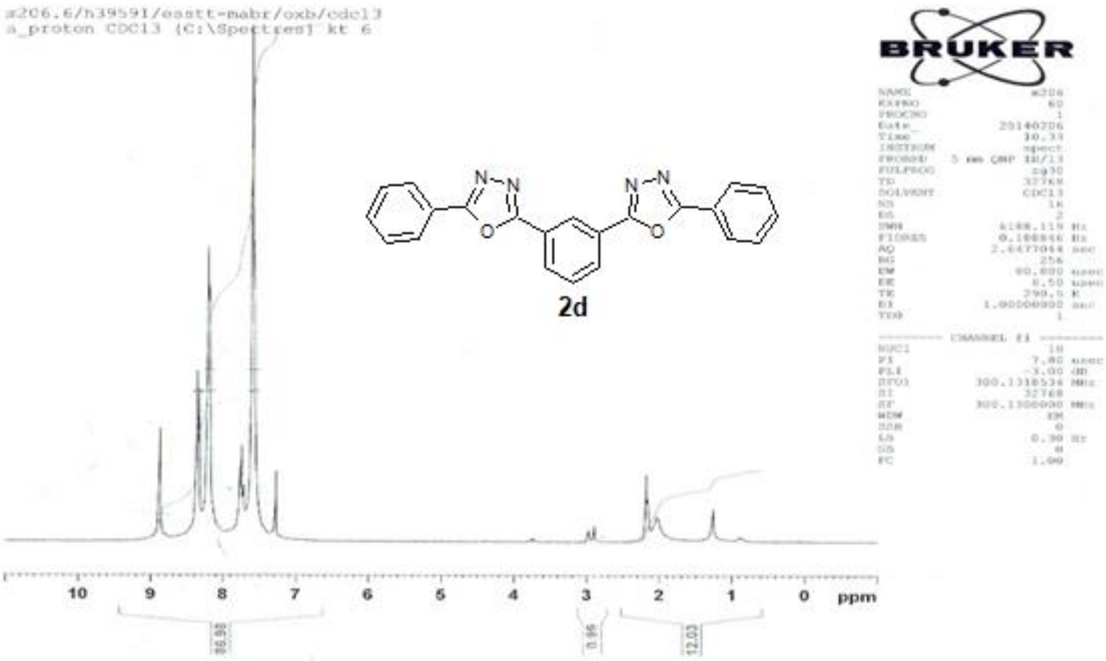

^{1}H NMR spectrum of compound **2d**.

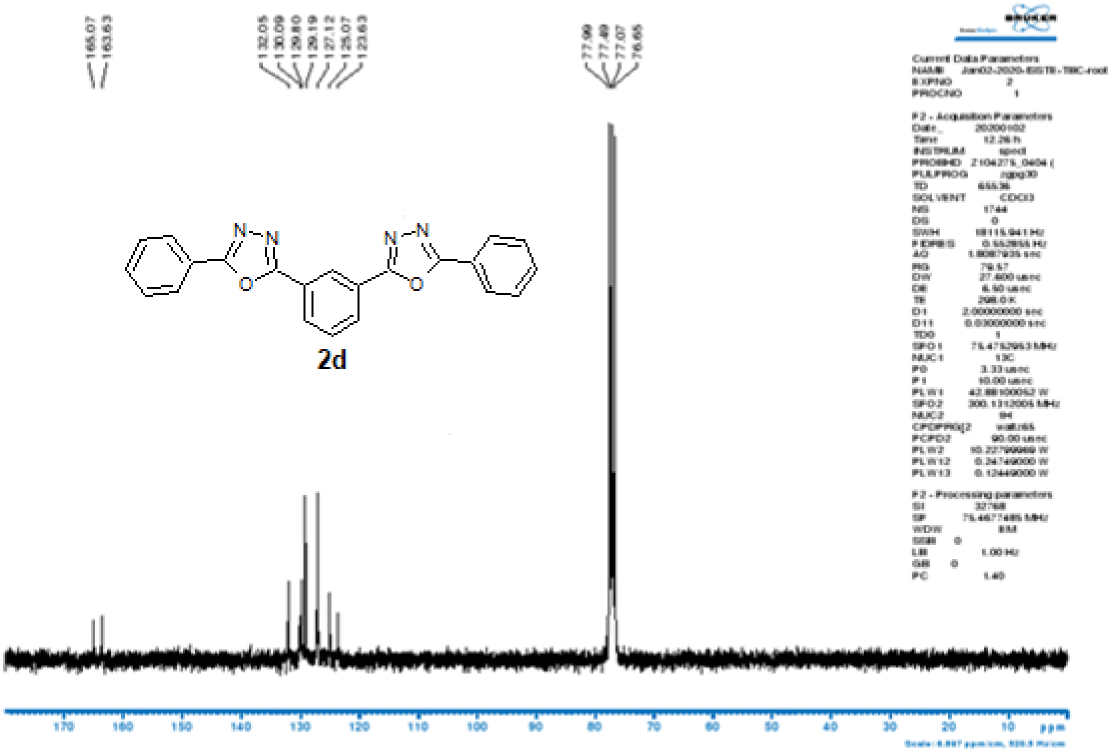

^{13}C NMR spectrum of compound **2d**.

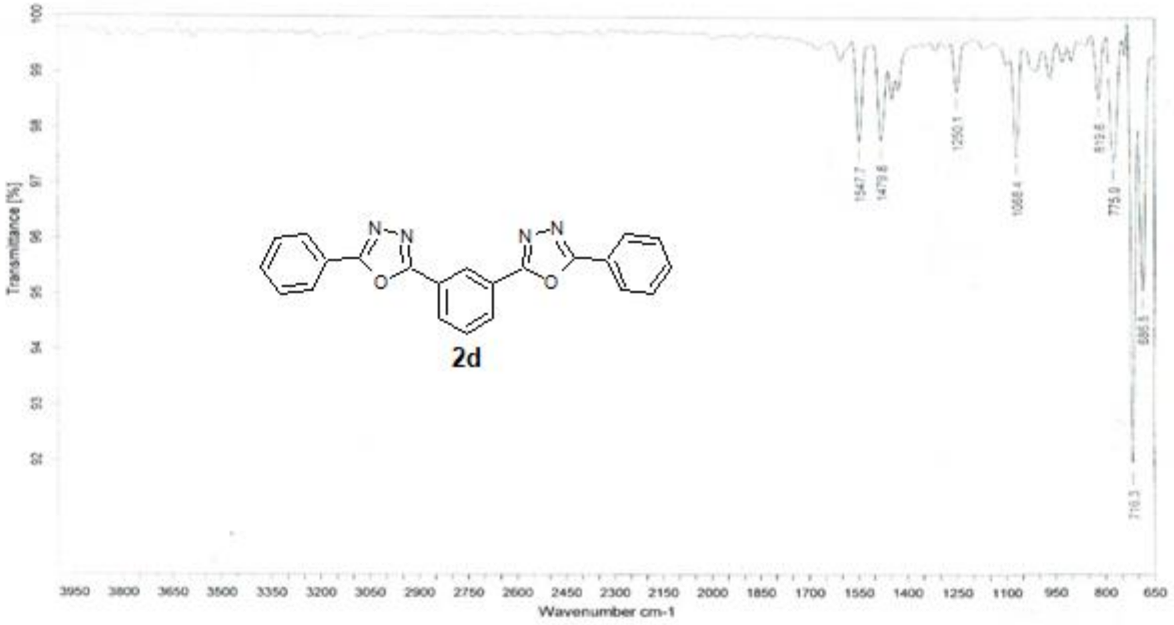

IR spectrum of compound **2d**.

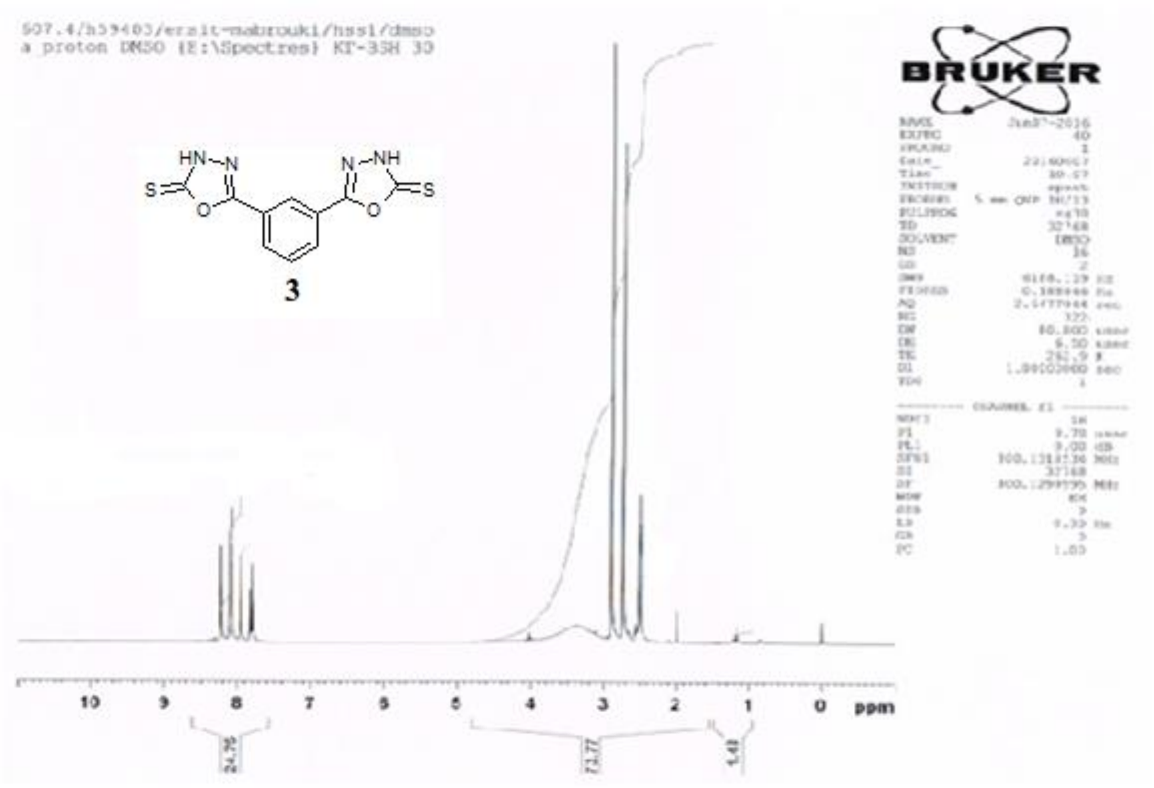

^{1}H NMR spectrum of compound **3**.

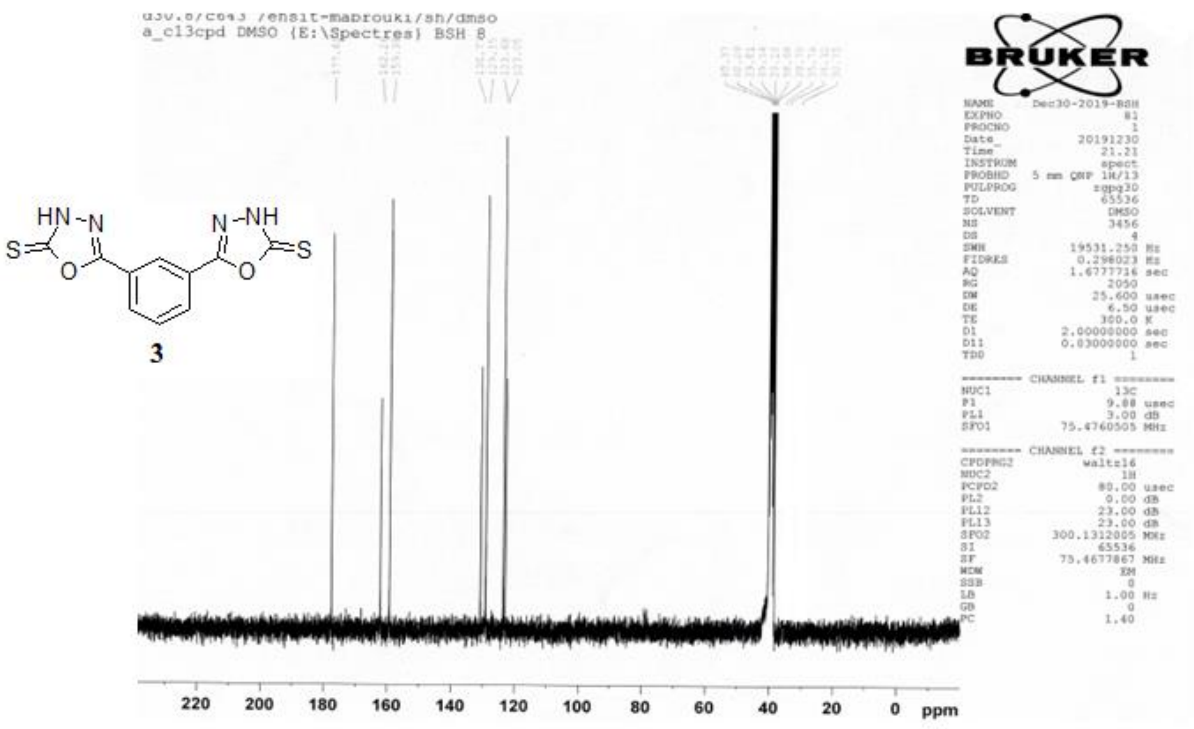

^{13}C NMR spectrum of compound **3**.

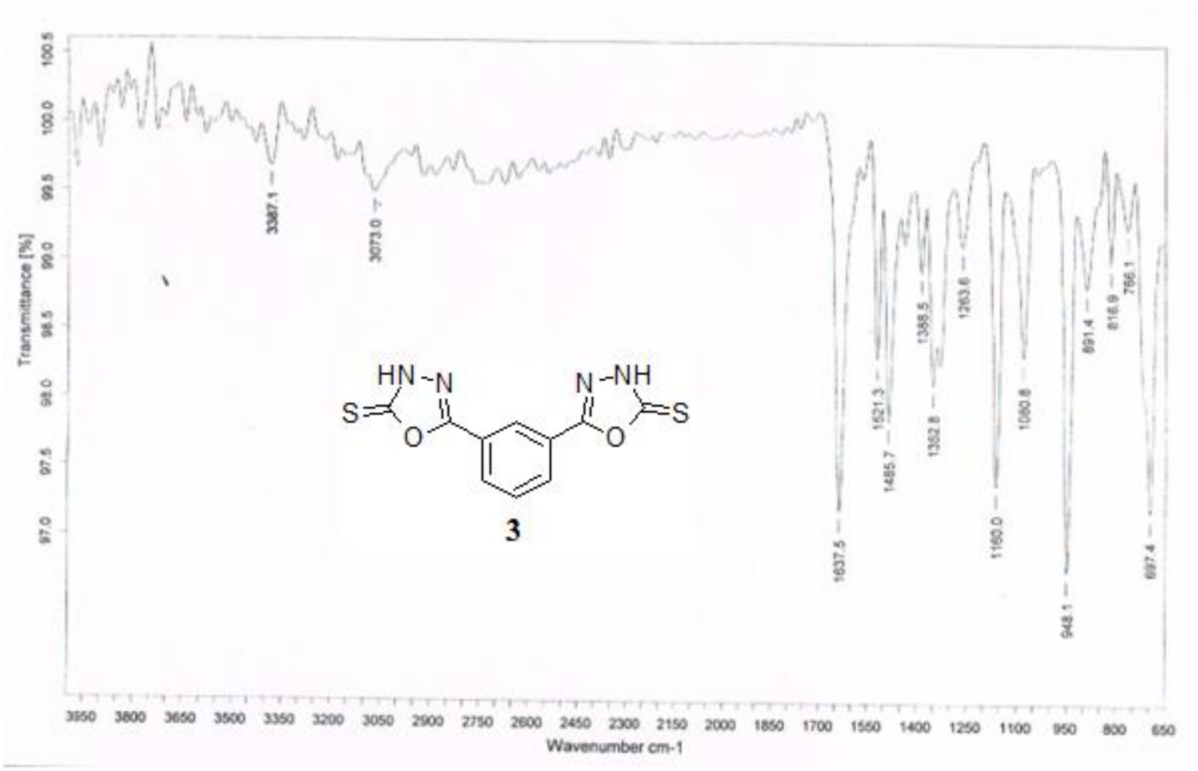

IR spectrum of compound **3**.

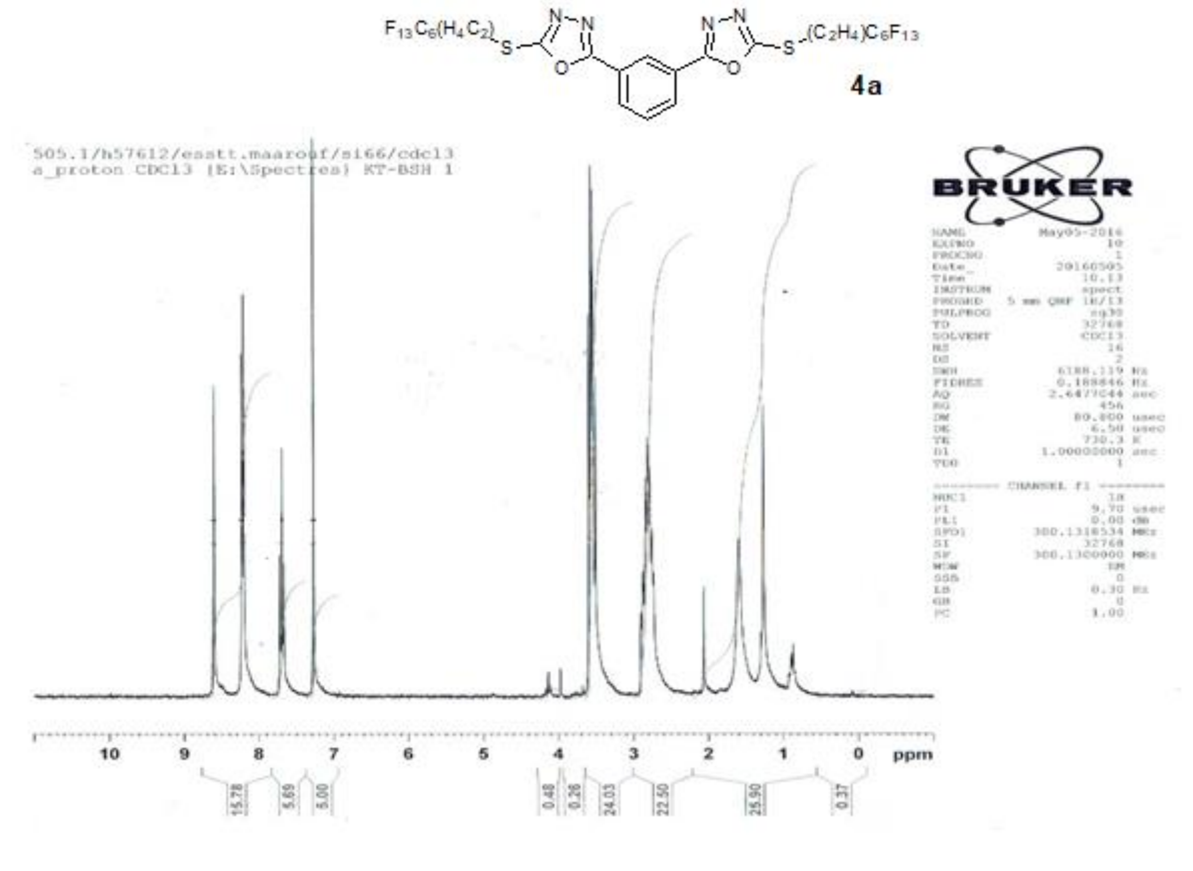

^{1}H NMR spectrum of compound **4a**.

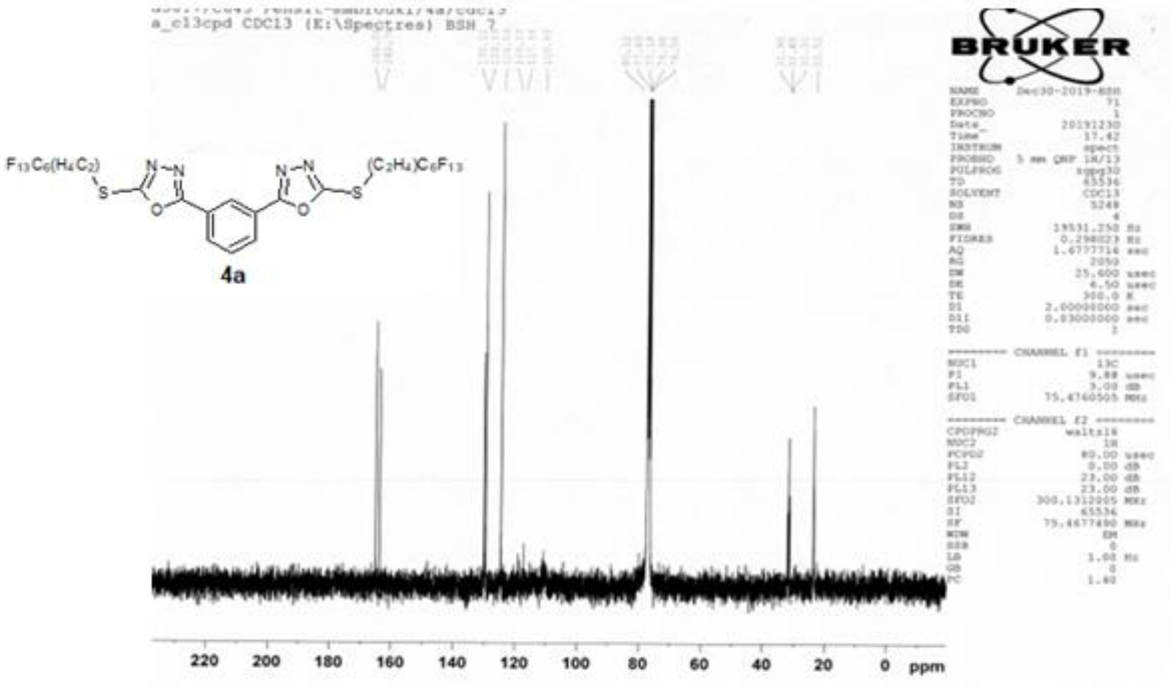

^{13}C NMR spectrum of compound **4a**.

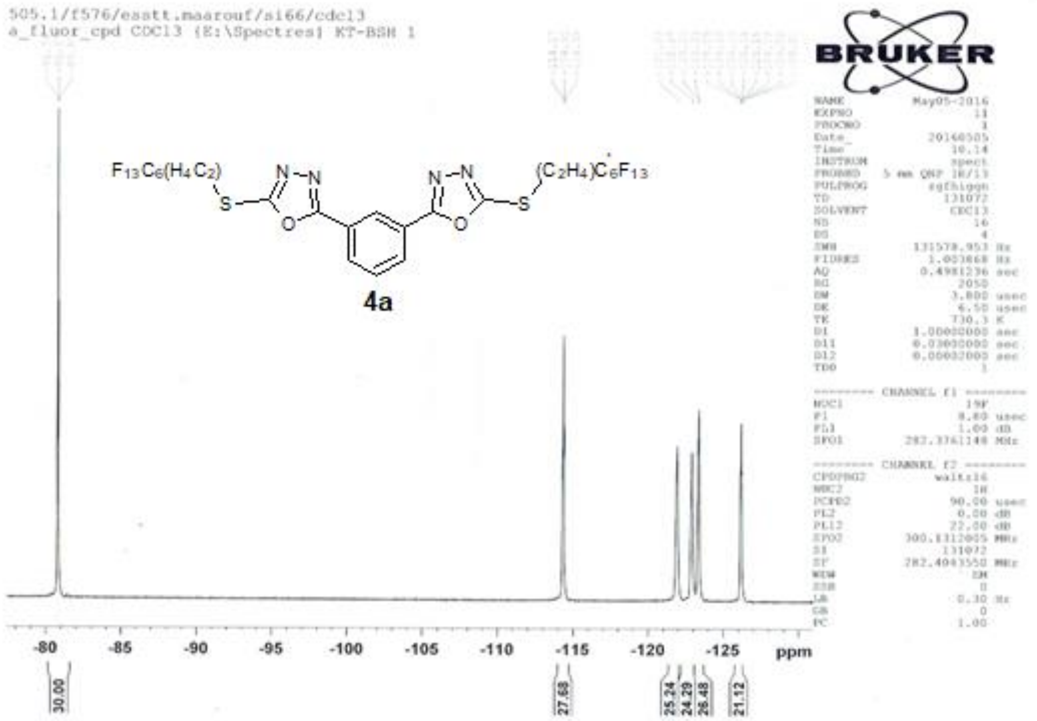

^{19}F NMR spectrum of compound **4a**.

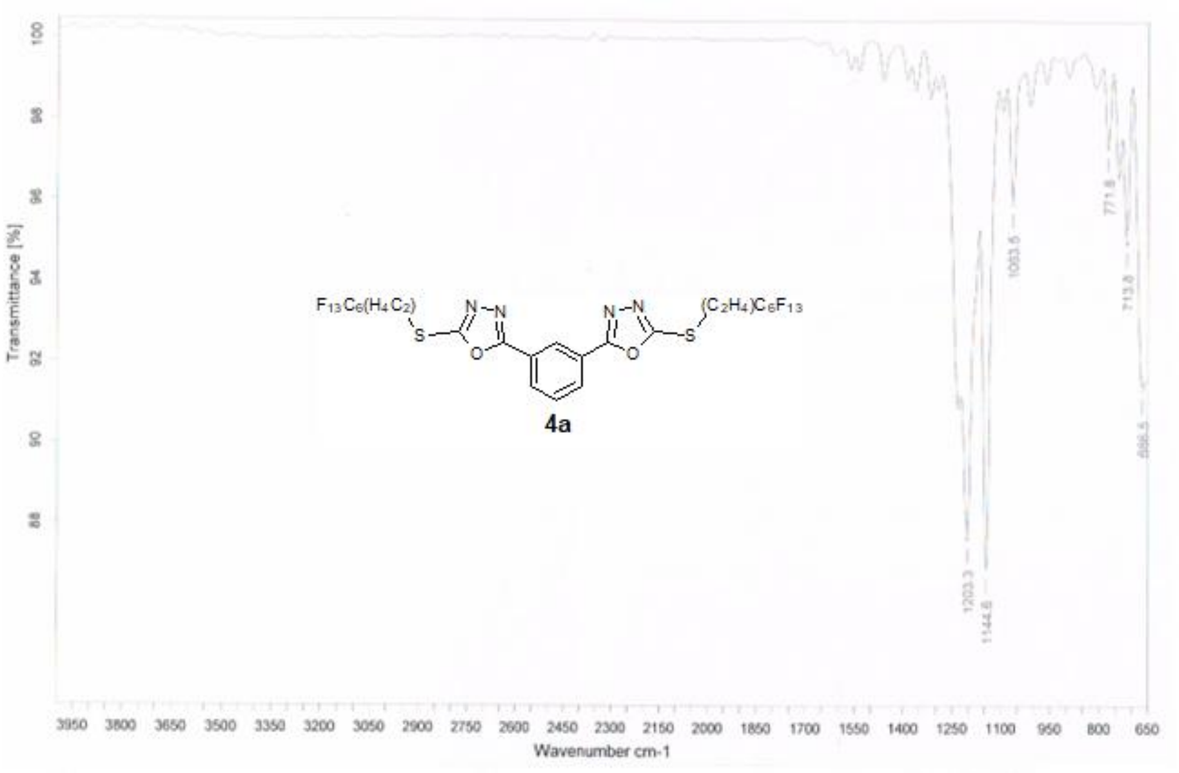

IR spectrum of compound **4a**.

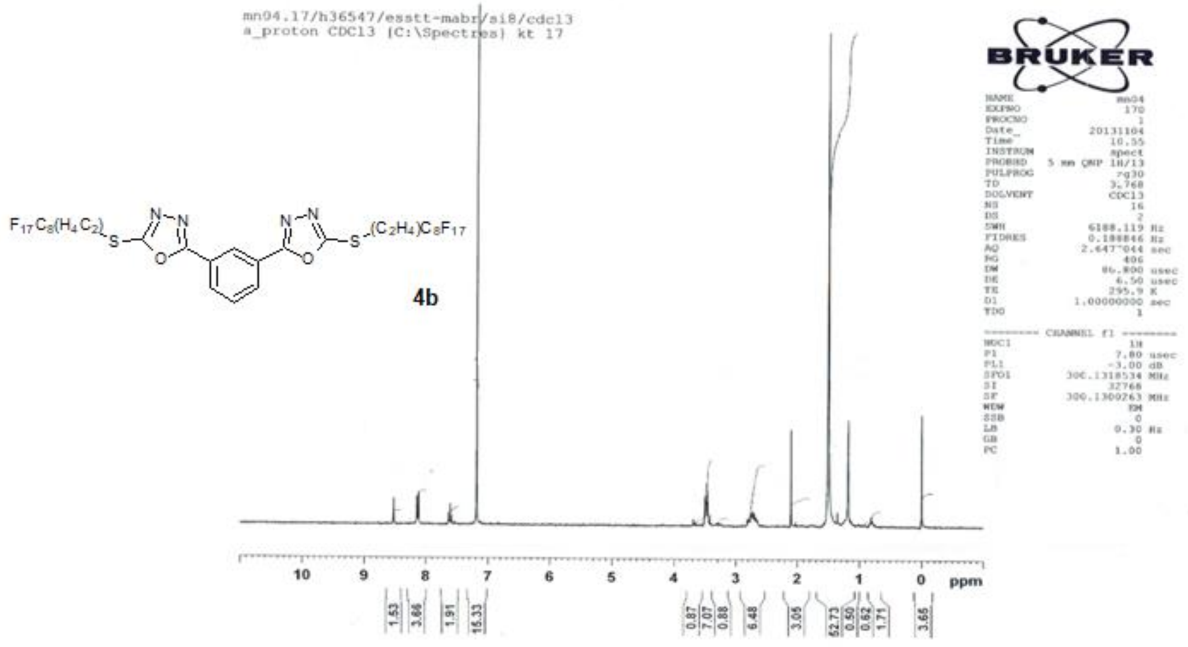

^{1}H NMR spectrum of compound **4b**.

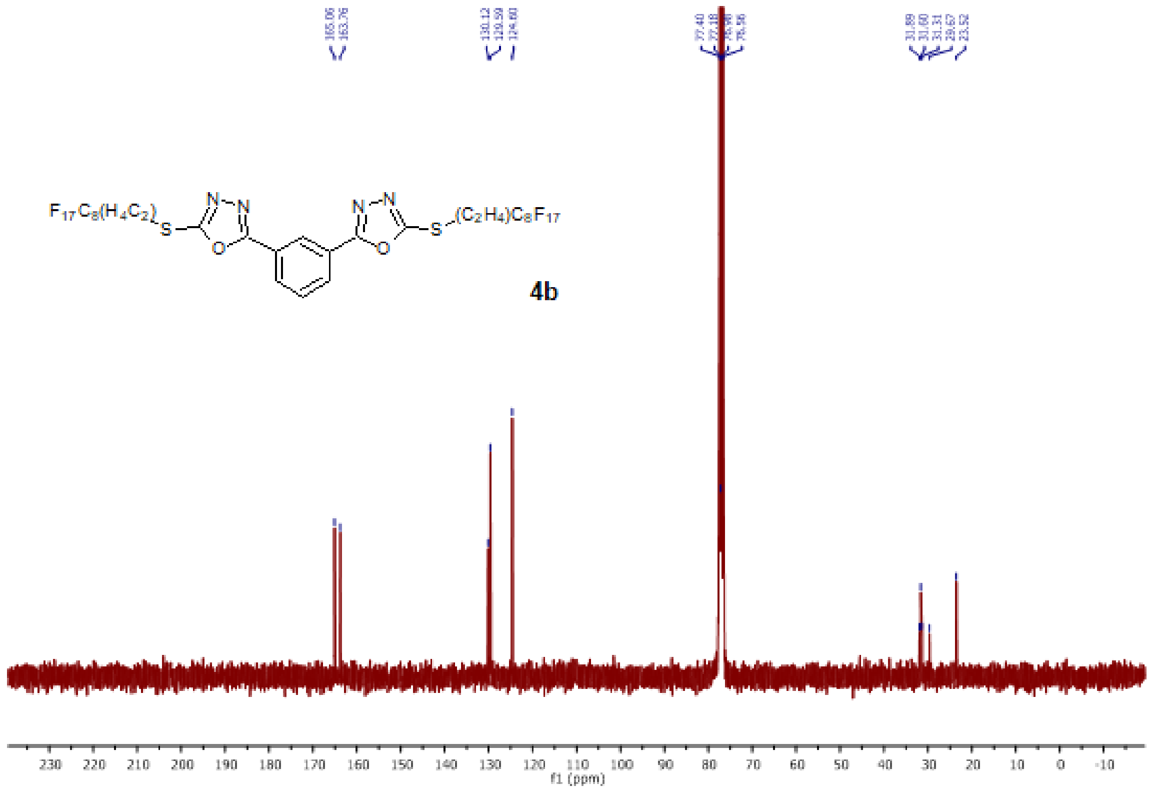

^{13}C NMR spectrum of compound **4b**.

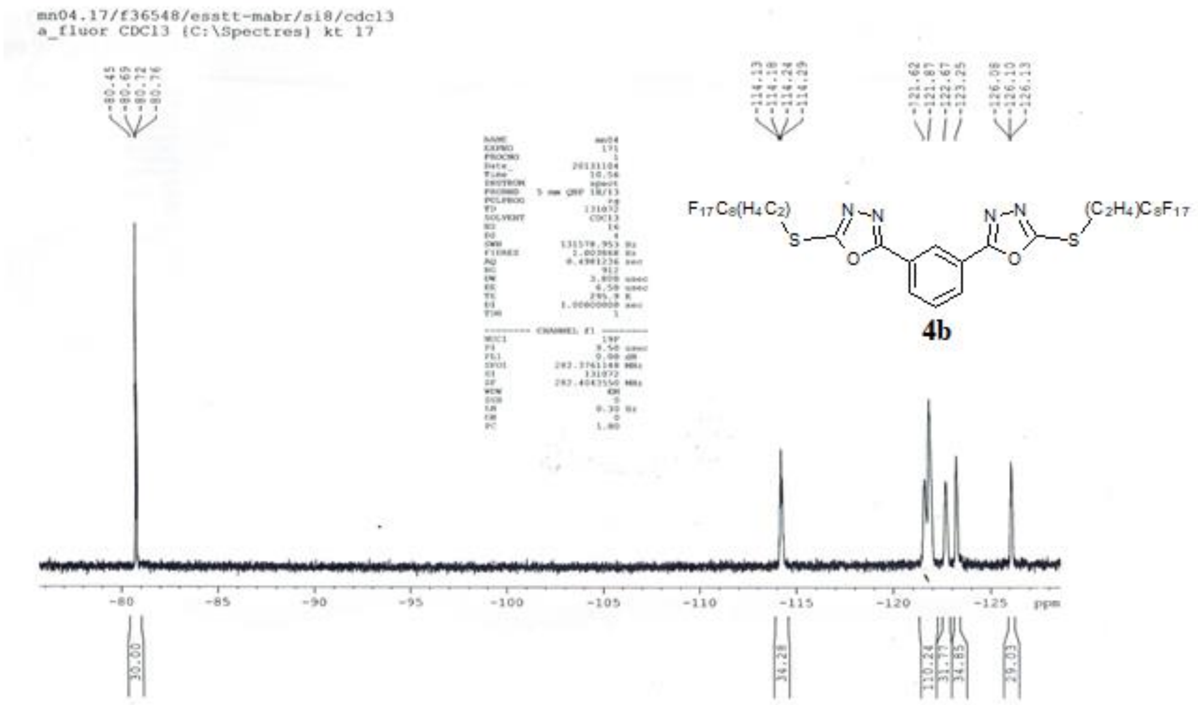

^{19}F NMR spectrum of compound **4b**.

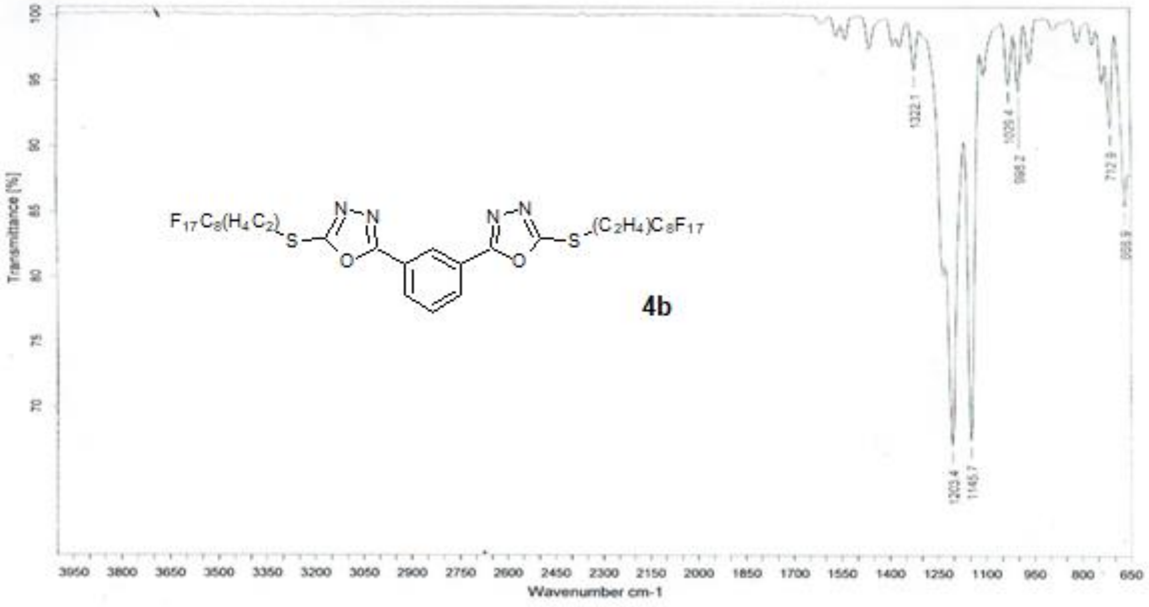

IR spectrum of compound **4b**.

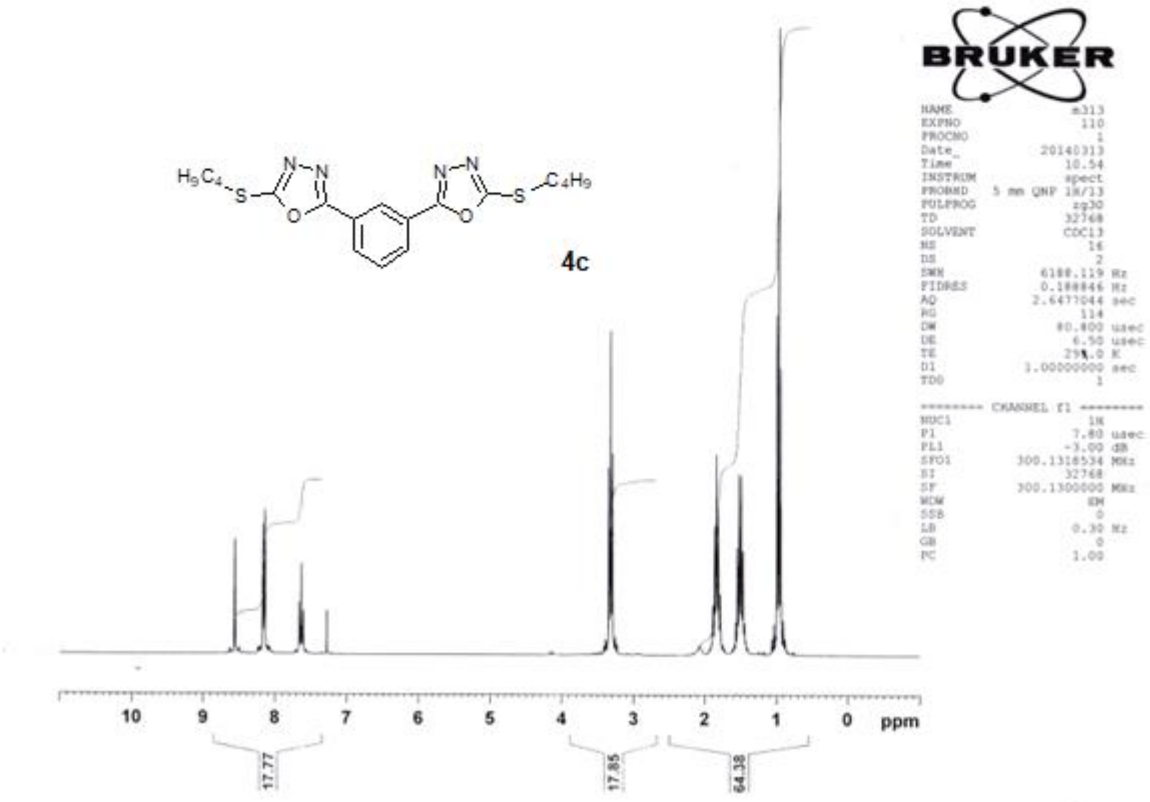

^{1}H NMR spectrum of compound **4c**.

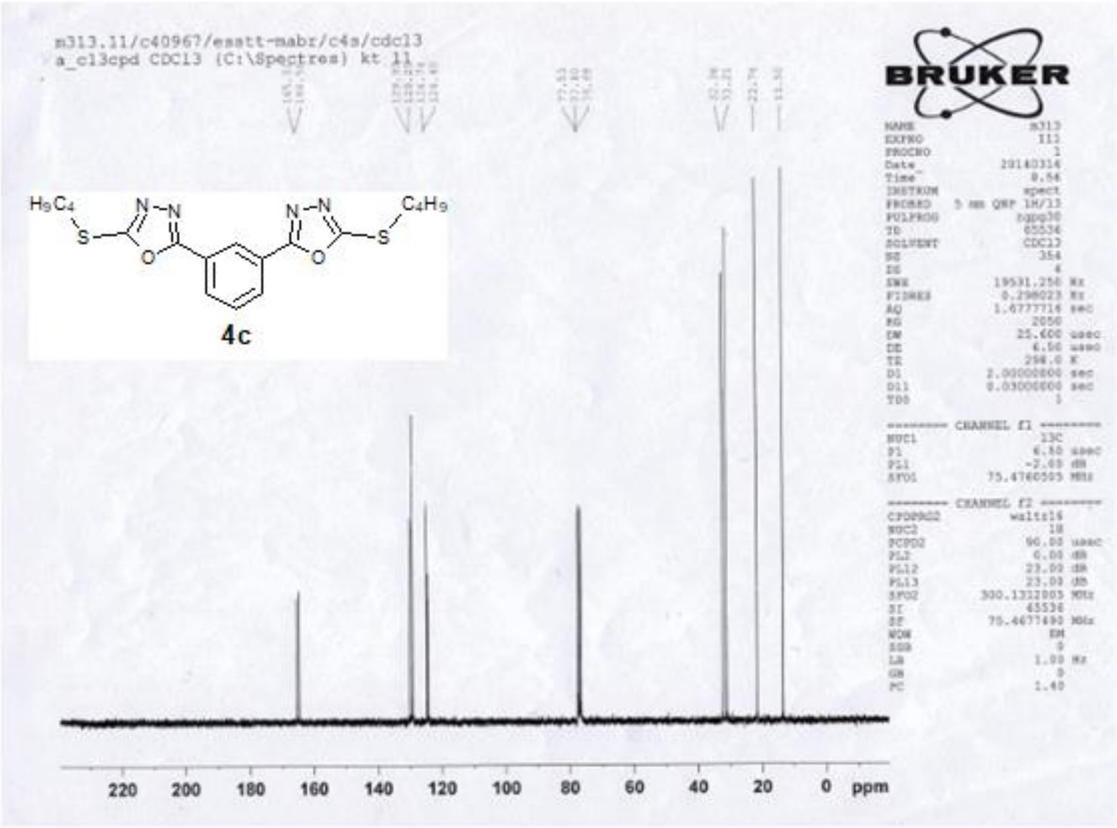

^{13}C NMR spectrum of compound **4c**.

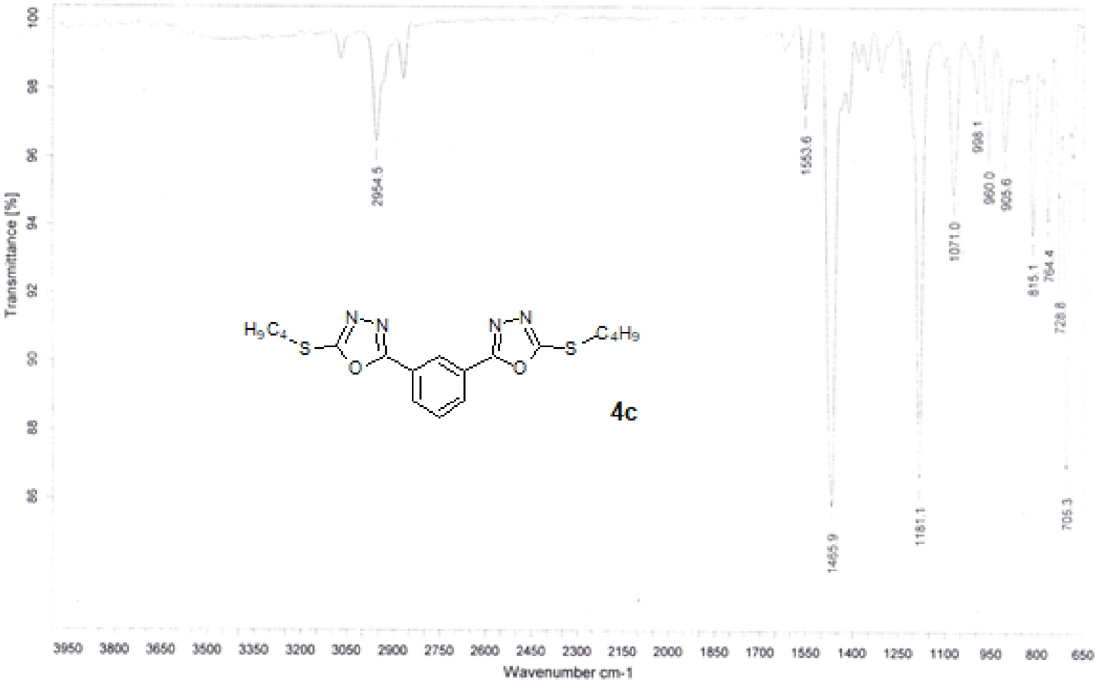

IR spectrum of compound **4c**.

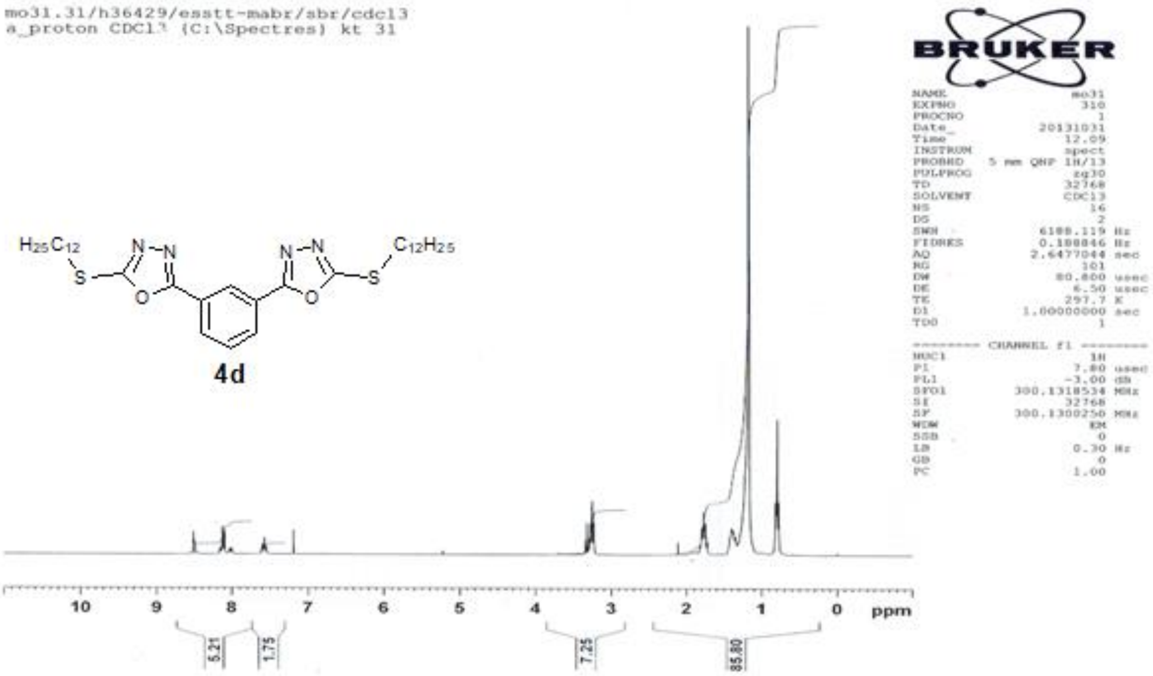

^{1}H NMR spectrum of compound **4d**.

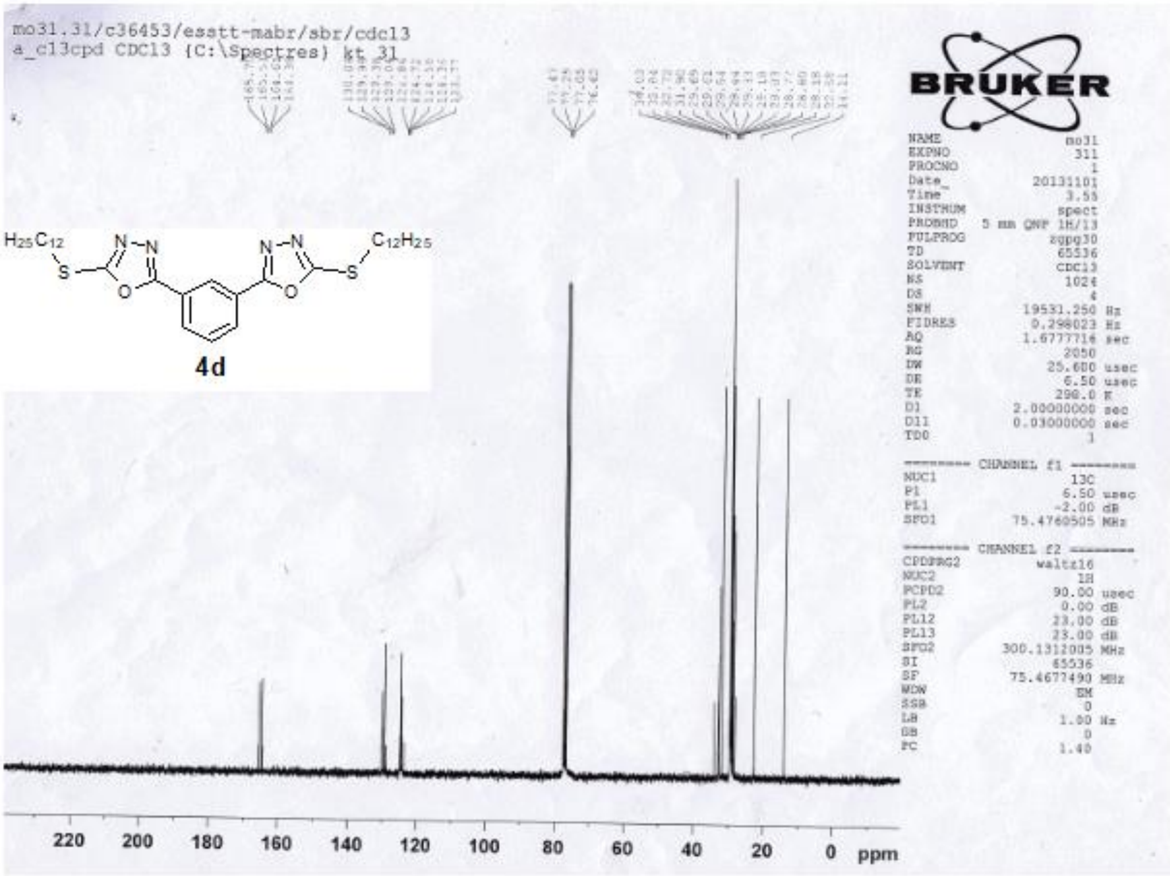

^{13}C NMR spectrum of compound **4d**.

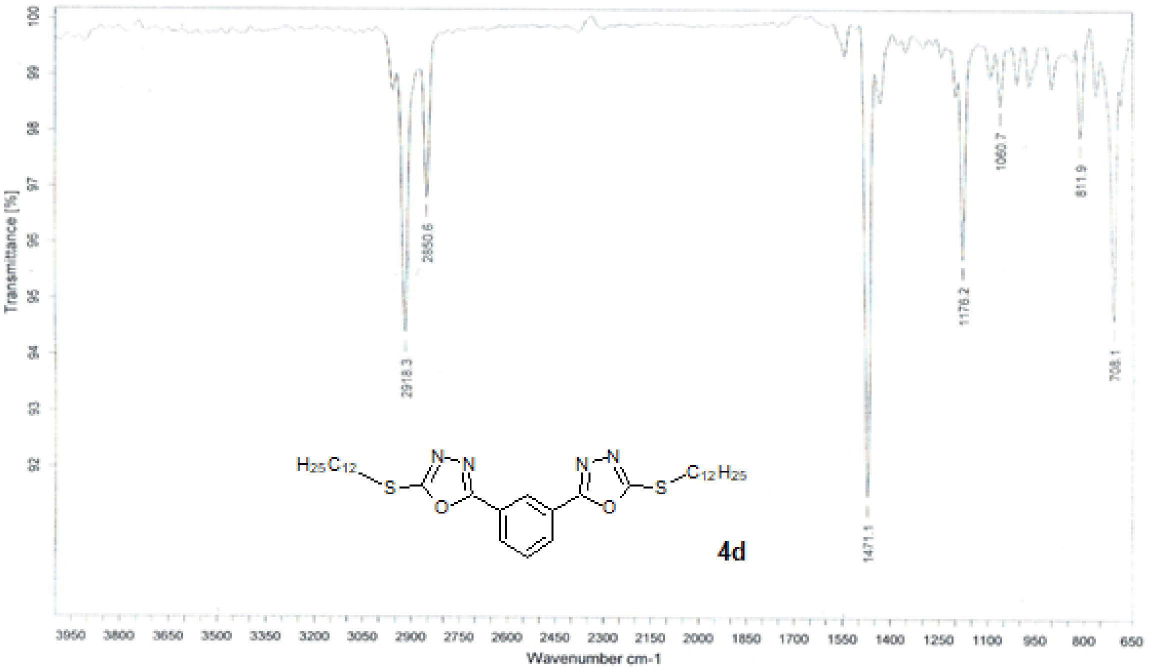

IR spectrum of compound **4d**.

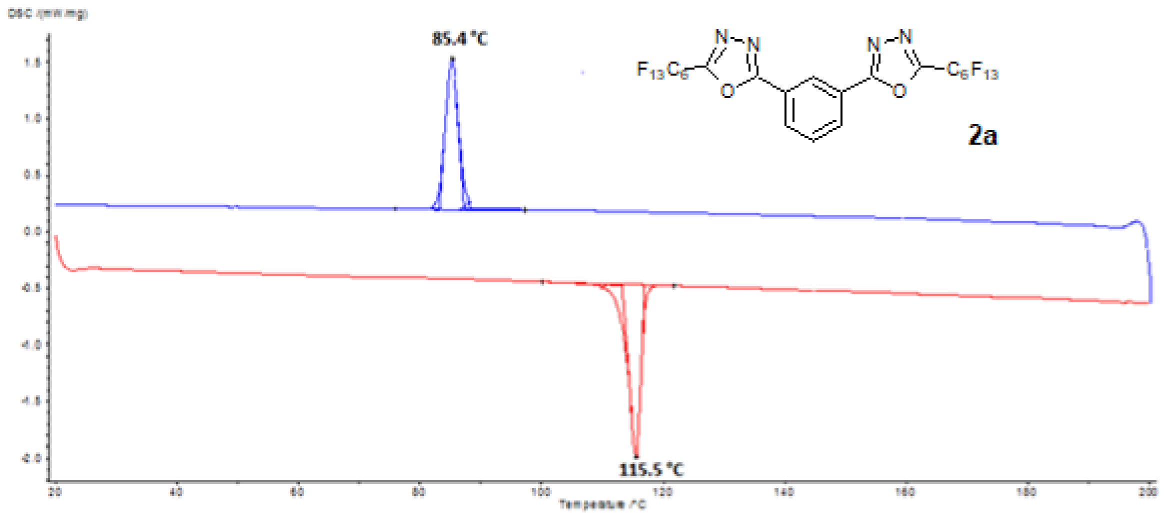

DSC thermogram of compound **2a** recorded at 5 °C/mn at heating (red) and cooling (blue) cycles.

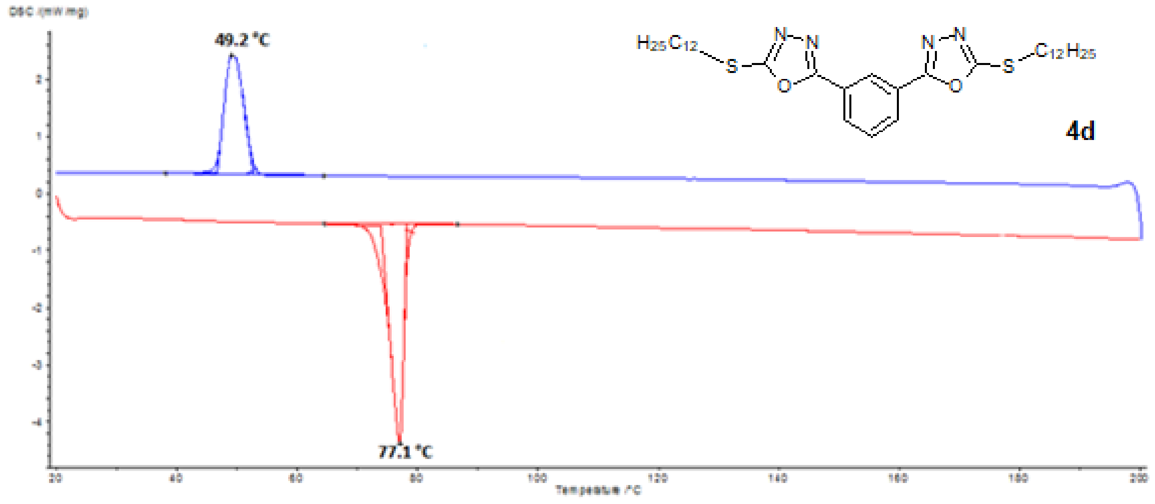

DSC thermogram of compound **4d** recorded at 5 °C/mn at heating (red) and cooling (blue) cycles.
